# IP_3_R1-mediated MAMs formation contributes to mechanical trauma-induced hepatic injury and the protective effect of melatonin

**DOI:** 10.1186/s11658-023-00509-x

**Published:** 2024-02-02

**Authors:** Rui Shi, Zhenhua Liu, Huan Yue, Man Li, Simin Liu, Dema De, Runjing Li, Yunan Chen, Shuli Cheng, Xiaoming Gu, Min Jia, Jun Li, Juan Li, Shumiao Zhang, Na Feng, Rong Fan, Feng Fu, Yali Liu, Mingge Ding, Jianming Pei

**Affiliations:** 1 Department of Geriatrics Cardiology, The Second Affiliated Hospital of Xi’an Jiaotong University, Xi’an, China; 2 Department of Physiology and Pathophysiology, National Key Discipline of Cell Biology, Fourth Military Medical University, Xi’an, China; 3 Key Laboratory of Surgical Critical Care and Life Support, Xi’an Jiaotong University, Ministry of Education, Xi’an, China; 4 School of Life Science, Northwest University, Xi’an, China; 5 The Key Laboratory of Shaanxi Province for Craniofacial Precision Medicine Research, Laboratory Center of Stomatology, Department of Orthodontics, College of Stomatology, Xi’an Jiaotong University, Xi’an, China

**Keywords:** Mechanical trauma, Melatonin, MAMs, ERK1/2, FoxO1, IP_3_R1

## Abstract

**Introduction:**

There is a high morbidity and mortality rate in mechanical trauma (MT)-induced hepatic injury. Currently, the molecular mechanisms underlying liver MT are largely unclear. Exploring the underlying mechanisms and developing safe and effective medicines to alleviate MT-induced hepatic injury is an urgent requirement. The aim of this study was to reveal the role of mitochondria-associated ER membranes (MAMs) in post-traumatic liver injury, and ascertain whether melatonin protects against MT-induced hepatic injury by regulating MAMs.

**Methods:**

Hepatic mechanical injury was established in Sprague–Dawley rats and primary hepatocytes. A variety of experimental methods were employed to assess the effects of melatonin on hepatic injury, apoptosis, MAMs formation, mitochondrial function and signaling pathways.

**Results:**

Significant increase of IP_3_R1 expression and MAMs formation were observed in MT-induced hepatic injury. Melatonin treatment at the dose of 30 mg/kg inhibited IP_3_R1-mediated MAMs and attenuated MT-induced liver injury in vivo. In vitro, primary hepatocytes cultured in 20% trauma serum (TS) for 12 h showed upregulated IP_3_R1 expression, increased MAMs formation and cell injury, which were suppressed by melatonin (100 μmol/L) treatment. Consequently, melatonin suppressed mitochondrial calcium overload, increased mitochondrial membrane potential and improved mitochondrial function under traumatic condition. Melatonin’s inhibitory effects on MAMs formation and mitochondrial calcium overload were blunted when IP_3_R1 was overexpressed. Mechanistically, melatonin bound to its receptor (MR) and increased the expression of phosphorylated ERK1/2, which interacted with FoxO1 and inhibited the activation of FoxO1 that bound to the IP_3_R1 promoter to inhibit MAMs formation.

**Conclusion:**

Melatonin prevents the formation of MAMs via the MR-ERK1/2-FoxO1-IP_3_R1 pathway, thereby alleviating the development of MT-induced liver injury. Melatonin-modulated MAMs may be a promising therapeutic therapy for traumatic hepatic injury.

**Graphical Abstract:**

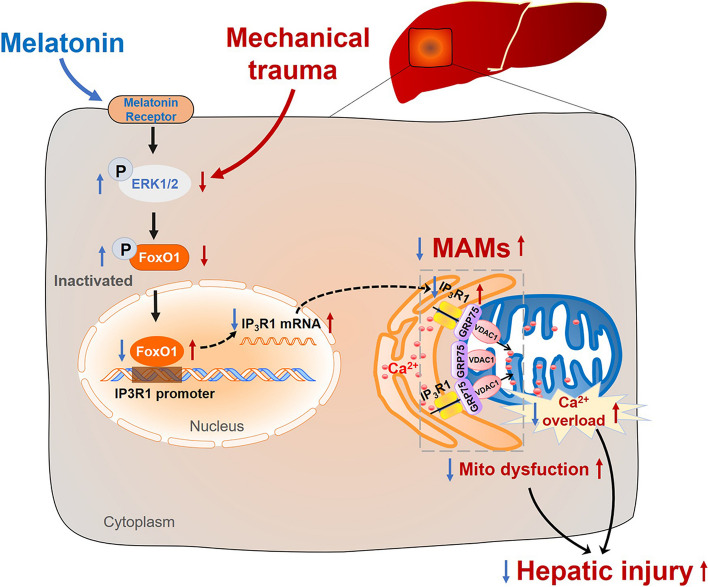

**Supplementary Information:**

The online version contains supplementary material available at 10.1186/s11658-023-00509-x.

## Introduction

Mechanical trauma (MT), caused by traffic accidents, sport injury and natural disasters, has been a common disease in emergency departments nowadays [1]. Mechanical trauma leads to multiple organ damages, among which abdominal trauma occurs mainly in the liver [2]. Besides the direct injury to the liver that occurs immediately after trauma (i.e. hemorrhage), several studies have indicated that MT often results in secondary hepatic injury in the later period of trauma [3–5]. It is difficult to diagnose secondary hepatic injury caused by MT accurately because its clinical features are often obscure. Secondary hepatic injury can present as serum biochemical abnormalities, increased inflammation and eventually hepatic failure [6–8]. Therefore, identifying the mechanisms responsible for post-traumatic hepatic injury and searching for effective therapeutic approaches are critical for MT patients.

Mitochondria are vital organelles as they not only provide ATP but also buffer calcium (Ca^2+^) and regulate cell apoptosis [9]. Endoplasmic reticulum (ER) is crucial for modifying newly synthesized proteins and regulating intracellular Ca^2+^ content [10]. According to recent studies, mitochondria and ER are not independent, but are actively communicating with each other through the contact sites, defined as mitochondria-associated ER membranes (MAMs) [11]. The tether complex primarily comprises three different types: ER-localized inositol 1,4,5-trisphosphate receptor (IP_3_R) and outer mitochondrial membrane protein voltage-dependent anion channel 1 (VDAC1) interact via chaperone glucose-regulated protein 75 (GRP75); ER-localized mitofusin 2 (Mfn2) interacts with mitochondrial Mfn1/2; ER-localized B-cell receptor-associated protein 31 (Bap31) binds to mitochondrial fission 1 protein (Fis1) [12–15]. Actually, MAMs have received increasing attention because of their important regulation of several key cellular activities, including the transport of ions and metabolites, mitochondrial dynamics, autophagy and inflammation [11, 16, 17]. Moreover, changes of MAMs structure or number result in the dysfunction of ER and mitochondrial and consequently lead to the development of multiple pathological conditions in liver, including hepatic insulin resistance, lipid metabolic imbalance, hepatotoxicity and hepatocyte death, while maintaining the integrity and number of MAMs improve the hepatocyte survival and function [18–22]. Although MAMs homeostasis have displayed a crucial role in several liver diseases, it remains unclear whether MAMs are involved in liver MT and its regulation mechanism.

Melatonin is mainly produced by the pineal gland and works in liver, intestine, and other organs as well, which plays pleiotropic roles in various physiological functions due to the ability of antioxidation, anti-inflammation, and anti-apoptosis [23, 24]. Melatonin has been reported to protect the liver from ischemia–reperfusion injury as well as has protective effects on liver fibrosis induced by multiple toxicants [25–29]. According to previous studies from our lab and others, melatonin exerts protective effects in blunt trauma-induced muscle and myocardial injury via modulating mitochondrial dynamics and anti-inflammatory cytokines [30, 31]. Considering melatonin's potential protective effects against liver disease and traumatic injury, we hypothesized that melatonin might protect against MT-induced liver injury.

Therefore, the main objectives of this study were (i) to explore the role of MAMs in post-traumatic liver injury; (ii) to ascertain whether melatonin has the potential to mitigate post-traumatic liver injury by regulating MAMs (iii) if so, to investigate how melatonin inhibits the adverse change of MAMs induced by MT?

## Materials and methods

### Animals and MT model

All animal experiments were conducted according to National Institutes of Health Guidelines on Animal Research and approved by the Fourth Military Medical University Animal Experiment Ethics Committee. Adult male Sprague–Dawley (SD) rats (190–230 g) were obtained from the Animal Experimental Center. Rats were anesthetized with 60 mg/kg sodium phenobarbital (i.p.) and placed in a Noble-Collip drum to induce MT model as previously described [32, 33]. The parameter was set to 5-min rotations at a rate of 40 rpm (200 revolutions) as we described previously [30]. The sham-traumatic rats were subjected to the same 5-min procedure but fixed on the inner wall of the drum to avoid MT injury. Traumatic rats were randomly assigned to receive one of the following treatments intraperitoneally 5 min after trauma: (i) DMSO as vehicle (1 mL/kg) and (ii) melatonin (M5250, Sigma-Aldrich, USA, 10 mg/kg or 30 mg/kg). The dosage of melatonin used was based on previous studies [30, 34]. Blood samples from sham or MT rats were centrifuged for 15 min at 3,000 rpm, and Sham serum (SS) or traumatic serum (TS) was collected and stored at − 80 °C. Serum alanine aminotransferase (ALT), aspartate aminotransferase (AST) (Solarbio, China), and T-AOC (total antioxidant capacity Assay) Assay Kits (Beyotime Biotechnology, China) were performed according to the manufacturer’s instructions.

### Quantification of MAMs

Liver samples were fixed in electron microscope fixation liquid overnight and then re-fixed in 1% osmium tetroxide for 1 h. After that, the fixed tissues were dehydrated in concentration gradients of acetone (30–100%), and finally embedded in 812 epoxy resin. After embedded and sliced, the tissue sections were double stained by uranium acetate and lead citrate and observed using a transmission electron microscope (JEOL 1230). MAMs were analyzed with Image-Pro Plus software (6.0). For analyzing the co-localization of ER and mitochondria in vitro, ER and mitochondria were marked by mTur-ER viruses (1.99*10^10^ PFU/ml, Green, Hanbio Technology Ltd, China) and MitoTracker Red CMXRos (100 nmol/L, Invitrogen, USA), respectively. Images were analyzed by Image J software [35].

### Histological analysis

HE staining was performed referring to the previous study [36]. Fresh liver tissues were fixed with 4% paraformaldehyde and embedded in paraffin. After being dewaxed in xylene and hydrated in gradient alcohol, tissue slices were stained with hematoxylin and eosin solution and visualized by microscope.

### Isolation of primary hepatocytes and appropriate serum concentration detected by CCK-8

Mice were exposed the portal vein after euthanasia and perfused with 100 mL of HEPES-buffered Tyrodes solution and then 80 mL type IV collagenase (Sigma, USA). Primary hepatocytes were released from the hepatic capsule and sedimented at 100 rpm for 5 min. Hepatocytes were planted in petri dishes pre-coated with rat tail collagen type I (Corning, USA) and cultured in medium at 37 °C and 5% CO_2_.

Hepatocytes were planted in 96-well plates with a density of 1 × 10^4^ cells/well for 8 h. In order to determine whether the injury factors were related to MT, the medium was then replaced by fresh medium supplemented with a series of rat serum concentration (5% SS, 10%SS, 15%SS, 20%SS, 30%SS, 50%SS, 5% TS, 10%TS, 15%TS, 20%TS, 30%TS, 50%TS) and cultured for 12 h. Then operations were conducted according to CCK-8 instructions (Dojindo, Japan). Primary hepatocytes were treated to 100 μmol/L melatonin (HY-B0075, MedChem Express, USA) with 20% SS or 20% TS for 12 h as previously described [30].

### Quantification of apoptosis, mitochondrial membrane potential (MMP) and mitochondrial oxidative stress

The tissues fixed in 4% paraformaldehyde were made into paraffin sections. Then, paraffin sections were dewaxed twice in xylene and washed once with a serial alcohol gradient. Subsequently, tissues were treated with proteinase K working solution and permeabilized through Triton-X100 solution. The tissues were stained with TUNEL reaction solution (Roche, Switzerland) according to the instructions.

Primary hepatocytes were obtained by enzymatic digestion (collagenase and accutase) and double stained by Annexin V/PI dye (40302ES20, Yeasen, China) according to manufacturer’s instructions. Changes in mitochondrial membrane potential (C2006, Beyotime Biotechnology, China) were also determined by flow cytometry analysis as previously described [37]. Hepatocytes were incubated with 5 mM MitoSOX solution (Invitrogen, USA) for 15 min and Images were analyzed by Image J software [38].

### Detection of mitochondrial Ca^2+^ concentration, cytosolic Ca^2+^ concentration and mitochondrial oxygen consumption rate (OCR)

The Rhod-2 AM dye (4 µM, 20 min, ab142780, Abcam, UK) and Fura-2 AM dye (2 µM, 20 min, ab120873, Abcam, UK) were used to detect the mitochondrial and cytosolic Ca^2+^ level, respectively. Images were obtained every 10 s, and Ionomycin (50402ES03, Yeasen, China) was added 60 s after the beginning of the observation [39]. The relative fluorescence intensity was measured by Image J software. OCR of hepatocytes was measured by using the XF24 Extracellular Flux Analyser (Agilent SeaHorse Bioscience, USA) as described previously [40].

### Adenovirus and siRNA transfection

Primary hepatocytes were transfected with empty adenoviral vectors (Ad-EV) or recombinant adenoviral vectors expressing IP_3_R1 (Ad-IP_3_R1) and FoxO1 (Ad-FoxO1). The titer of the adenoviruses used in this study was about 1.26*10^10^ PFU/ml (Hanbio Technology Ltd, China). ERK1/2 siRNA (sc-29308, sc-35336, Santa Cruz, USA), IP_3_R1 siRNA (sc-42476, Santa Cruz, USA) or negative control siRNA (sc-37007, Santa Cruz, USA) were transfected to hepatocytes using OPTI-MEM (Invitrogen, USA) and Lipofectamine RNAi MAX reagent (Invitrogen, USA) respectively.

### Quantitative real-time (RT)-PCR and Chromatin immunoprecipitation (ChIP) analysis

Total RNA was extracted from liver tissue or hepatocytes using Trizol, and then was reverse transcribed to cDNA using reverse transcription kits (#RR036A, Takara, Japan). Quantitative RT-PCR was performed on the Bio-Rad Real-Time PCR System. Supplementary Table 1 contained a list of all the primer sequences. ChIP assay was carried out using the SimpleChIP Plus Enzymatic Chromatin IP kit (#9005; Cell Signaling Technology, USA) according to the manufacturer’s instructions as described previously [41]. Additional file 2: Table S1 contained the sequences of the IP_3_R1 promoter region primers. IgG was employed as the negative control.

### Western blotting and co-immunoprecipitation (Co-IP)

RIPA buffer (Beyotime Biotechnology, China) containing protease inhibitor cocktail was used to extract total protein from liver tissue and hepatocytes. The standard Western blotting method was used as previously described [42]. Co-IP assay was carried out using a Pierce Classic Magnetic IP/Co-IP Kit (#88804, Thermo Fisher Scientific, USA) according to manufacturer’s instructions as described previously [43]. Co-IP products were then run for western blotting. Additional file 2: Table S2 contained a list of all the antibodies used.

### Luciferase activity assay

The luciferase activity assay was carried out using the Dual-Luciferase Reporter Assay System (Promega, USA) as directed by the manufacturer’s instructions. HEK-293 T cells were transfected with plasmids coding for pGL-IP_3_R1-promoter [− 2000/ + 0 bp] or pGL-Basic using lipofectamine 2000 (Invitrogen, USA), and then infected with plasmid containing FoxO1, JUNB, MEF2A, MEF2C, Nfatc2 or an empty vector. Luciferase activity was evaluated 48 h after infection.

### Statistical analysis

All data in the experiment are expressed as the mean ± SEM. All statistical analyses were carried out with GraphPad Prism 8.0 software using One-way analysis of variance (ANOVA) or two-way ANOVA followed by Turkey or Bonferroni’s multiple comparison test where appropriate. *P* values < 0.05 were considered statistically significant.

## Results

### Significant increase of MAMs was observed in MT-induced hepatic injury

**Fig. 1:**
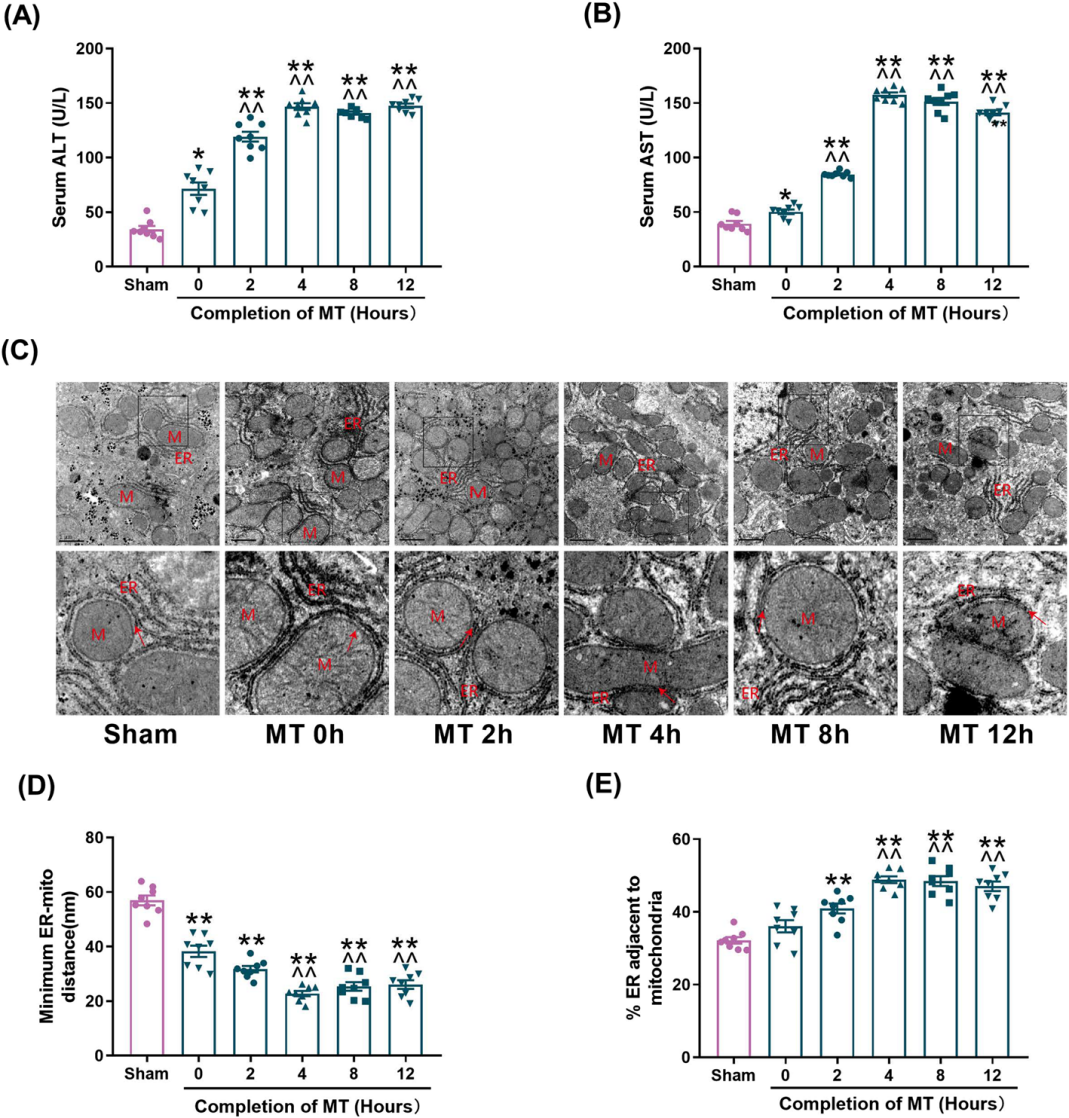
Significant increase of MAMs was observed in MT-induced hepatic injury. **A** Serum ALT levels at different time points. **B** Serum AST levels at different time points. **C** Representative TEM images at different time points at × 15,000 magnification. Scale bars: 1 μm. Pictures in bottom side are the magnified ones from top side, indicated by black frame. M: mitochondria; ER: Endoplasmic reticulum; Red arrow: minimum ER-mitochondria distance. **D** Quantification of the minimum distance between ER and mitochondria. **E** Quantification of ER length close to mitochondria normalized by total ER length at different time. All of the values are shown as the means ± SEM. *n* = 8 in each group. *P < 0.05, **P < 0.01 vs Sham; ^^^^*P* < 0.01 vs 0 h after MT

As shown in Fig. 1A, B, ALT, AST were immediately increased in MT rats after 5-min trauma (time 0 h) and gradually increased thereafter. Moreover, levels of AST and ALT were significantly increased in the serum of 4-12 h groups as compared with the 0 h group after MT, which suggested that both direct and secondary liver injury were caused by MT and secondary liver injury occurred predominantly after 4 h of MT.

In order to investigate ER and mitochondria interaction in MT, transmission electron microscopy was utilized to evaluate the changes of MAMs. As shown in Fig. 1C–E, the minimum distance between ER and mitochondria was significantly reduced after MT and reached the lowest point at 4 h after MT. The ratio of ER close to mitochondria to total ER length was not increased immediately after MT (time 0), but gradually increased thereafter after 2 to 12 h, which indicated that the formation of MAMs was increased. These results suggested that MT-induced hepatic injury was accompanied by the increase of MAMs.

### Melatonin attenuated MT-induced liver damage and cell death

**Fig. 2:**
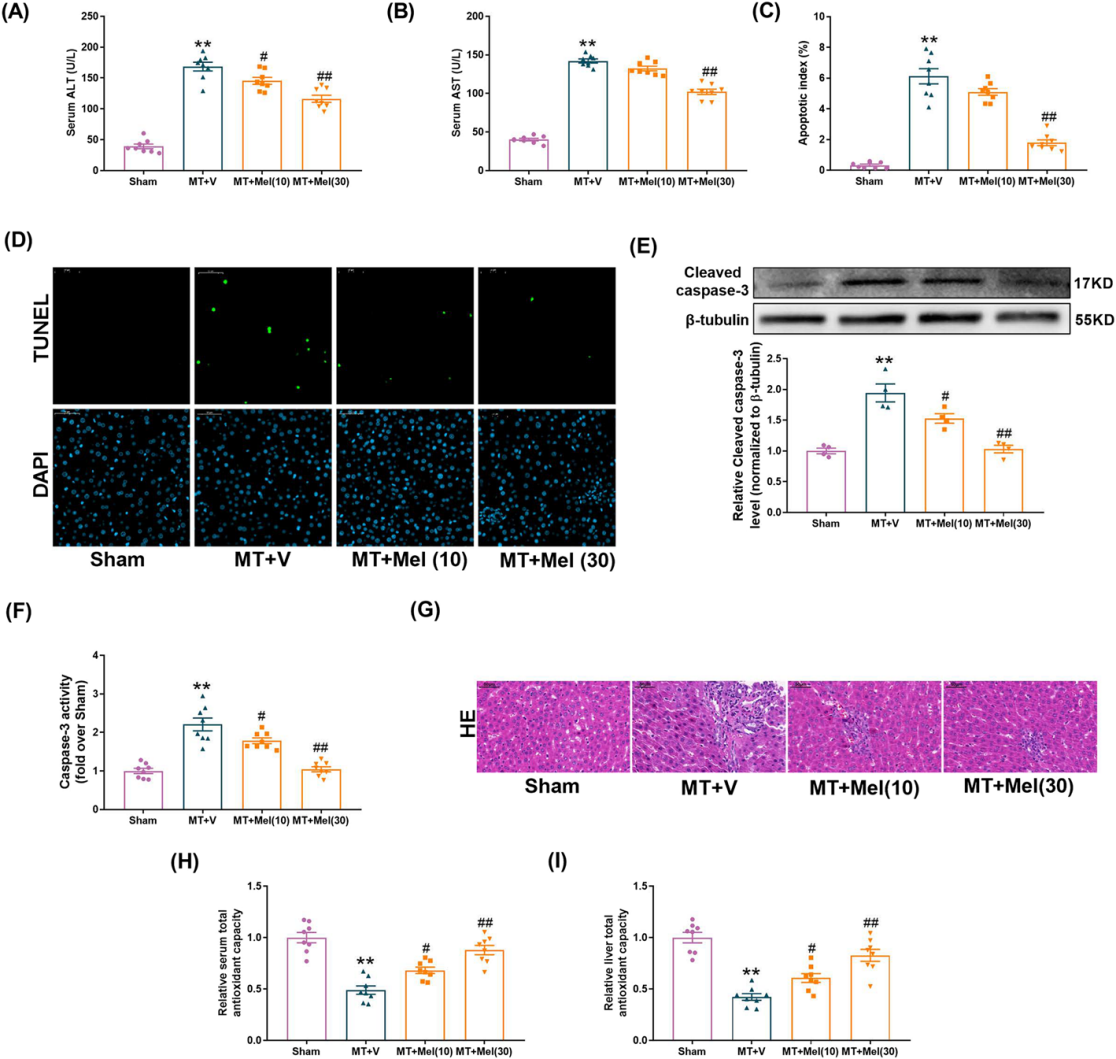
Melatonin attenuated MT-induced liver damage and apoptosis in vitro. **A** The levels of ALT release (fold difference compared with SS). **B** The levels of AST release (fold difference compared with SS). **C** Apoptosis index. **D** Representative TUNEL and DAPI-stained liver sections at × 400 magnification. **E** Representative blots and quantitative analysis of Cleaved-caspase 3 (*n* = 4). **F** Relative activity of caspase 3. **G** Representative liver HE images. **H** Total Antioxidant capacity of serum. **I** Total Antioxidant capacity of liver. All of the values are shown as the means ± SEM. *n* = 8 in each group. **P < 0.01 vs Sham; ^#^*P* < 0.05, ^##^*P* < 0.01 vs MT + V

The animals were administrated with 10 mg/kg or 30 mg/kg (i.p.) melatonin to determine whether melatonin served to protect the liver against MT-induced hepatic injury (4 h after MT). As shown in Fig. 2A, the level of ALT was significantly reduced by melatonin at the dose of 10 mg/kg (*P* < 0.05) and 30 mg/kg (*P* < 0.01). The inhibition of serum AST after MT occurred at the dose of 30 mg/kg rather than 10 mg/kg (Fig. 2B). Meanwhile, there was an increase in hepatocyte apoptosis (as measured by the apoptosis index, the expression of cleaved caspase-3 and caspase 3 activity) at 4 h after MT in comparison with sham group (Fig. 2C–F). Different from the complete structure of liver cells in the sham group, cell necrosis and many inflammatory cell infiltrations were detected after MT (Fig. 2G). The serum and liver antioxidant capacity decreased after MT (FIg. 2H, I), which were significantly mitigated by melatonin (30 mg/kg, i.p.) (Fig. 2C–I). These results suggested that treatment of melatonin at the dose of 30 mg/kg alleviated the hepatic injury and decreased the cell apoptosis in the MT rats.

### Melatonin inhibited IP_3_R1 elevation and suppressed MAMs formation in the liver following MT

**Fig. 3:**
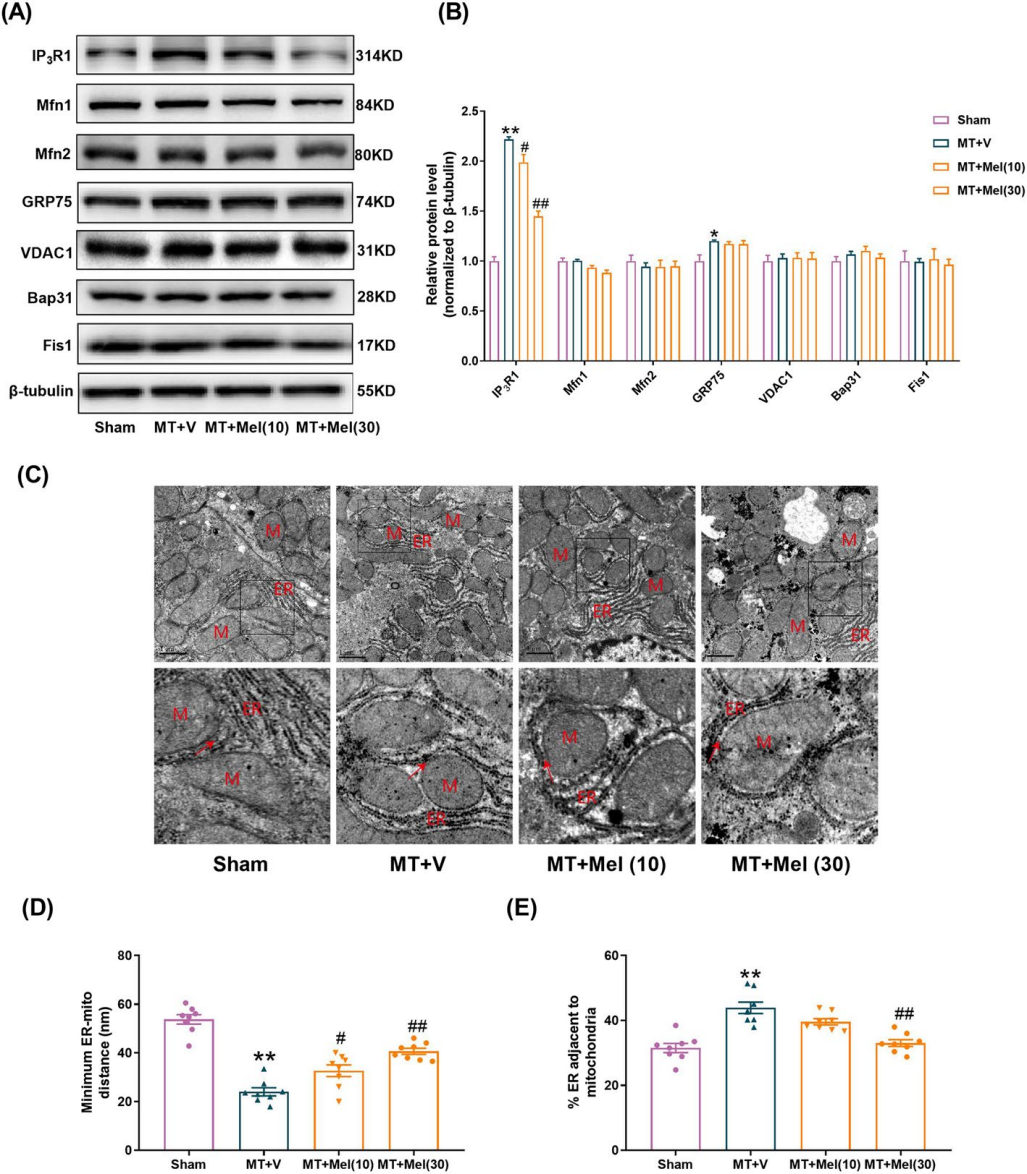
Melatonin inhibited IP_3_R1 elevation and suppressed MAMs formation in the liver following MT. **A**, **B** Representative blots and quantitative analysis of representative functional proteins (VDAC1, IP_3_R1, GRP75), structural proteins (Mfn1, Mfn2) and apoptotic related protein (Bap31, Fis1) of MAMs (*n* = 4). **C** Representative TEM images at × 15,000 magnification. Scale bars: 1 μm. **D** Quantification of the minimum distance between ER and mitochondria. **E** Quantification of ER length close to mitochondria normalized by total ER length. All of the values are shown as the means ± SEM. *n* = 8 in each group. *P < 0.05, **P < 0.01 vs Sham; ^#^*P* < 0.05, ^##^*P* < 0.01 vs MT + V

A range of main proteins related to the MAMs structure were then detected by western blotting in the liver at 4 h after MT. There were no significant changes in the expressions of Mfn1, Mfn2, VDAC1, Bap31 and Fis1 in the MT + V (Vehicle) group versus the Sham group (Fig. 3A, B). Importantly, IP_3_R1 was significantly increased, and GRP75 was slightly increased after MT, which suggested that IP_3_R1 was mainly involved in MAMs formation in the hepatic injury induced by MT. Moreover, we found that IP_3_R1 and MAMs formation were significantly decreased by melatonin at the dose of 30 mg/kg (Fig. 3A–E). These results suggested that treatment of melatonin (30 mg/kg) inhibited the rise of IP_3_R1 and suppressed MAMs formation in the liver subjected to MT.

### Melatonin inhibited cell death and MAMs formation in the hepatocytes cultured in TS

To explore the origin of the pathological mediators presented in the blood of traumatic animals or in traumatic hepatocytes themselves during MT hepatic injury, the hepatocytes were isolated from either sham traumatic animals [normal hepatocytes (NH)] or traumatic animals [traumatic hepatocytes (TH)] after MT and cultured in vitro for 12 h with medium containing 20% serum obtained from sham (sham serum, SS) or trauma animals (traumatic serum, TS; isolated from the MT animals) (Additional file 1: Fig. S1A). As shown in Additional file 1: Fig. S1B and 1C, the addition of TS to NH or TH caused a significant increase in cellular apoptosis, while no significant cellular apoptosis was observed when TH were cultured with SS. These results indicated that the mediators that cause hepatic injury after MT were mainly present in the serum of traumatic animals and not in TH themselves. Next, to identify the optimal concentration of TS or SS for in vitro as the MT model, a range of diluted concentrations were administered to the cells. As shown in Additional file 1: Fig. S1D, there were no noticeable changes in cell viability between cells with different concentrations of SS and control cells with normal medium, while evident changes occurred in the cells with TS (10%, 15%, 20%, 30% and 50%) compared with control cells. It was in 20% diluted TS that the cellular viability reduced close to 50%, which suggested 20% TS might be the appropriate concentration for MT in vitro model.

Previous studies from our lab and others have shown that the pro-inflammatory cytokines TNF-α, IL-1β and IL-6 were significantly elevated in serum after trauma and TNF-α played a pivotal role in MT-induced cardiac injury [29, 30]. TNF-α, IL-1β and IL-6 were examined in this study. Obviously, 3 main kinds of cytokines increased after MT and reached their highest level before or at 2 h after MT, ahead of the secondary hepatic injury happened (Additional file 1: Fig. S1E-G). We supposed whether one or more of these cytokines were associated with the cell death induced by TS. As shown in Additional file 1: Fig. S1H, not only the mixture of 3 cytokines (IL-1β: 1 ng/mL, IL-6: 1 ng/mL, TNF-α: 10 ng/mL) but also only TNF-α (10 ng/mL) significantly resulted in cell death similar to that induced by TS, which indicated that serum TNF-α led to hepatocyte injury induced by TS. Moreover, treatment of melatonin after MT significantly prevented the elevation of TNF-α in serum (Additional file 1: Fig. S1I).

**Fig. 4:**
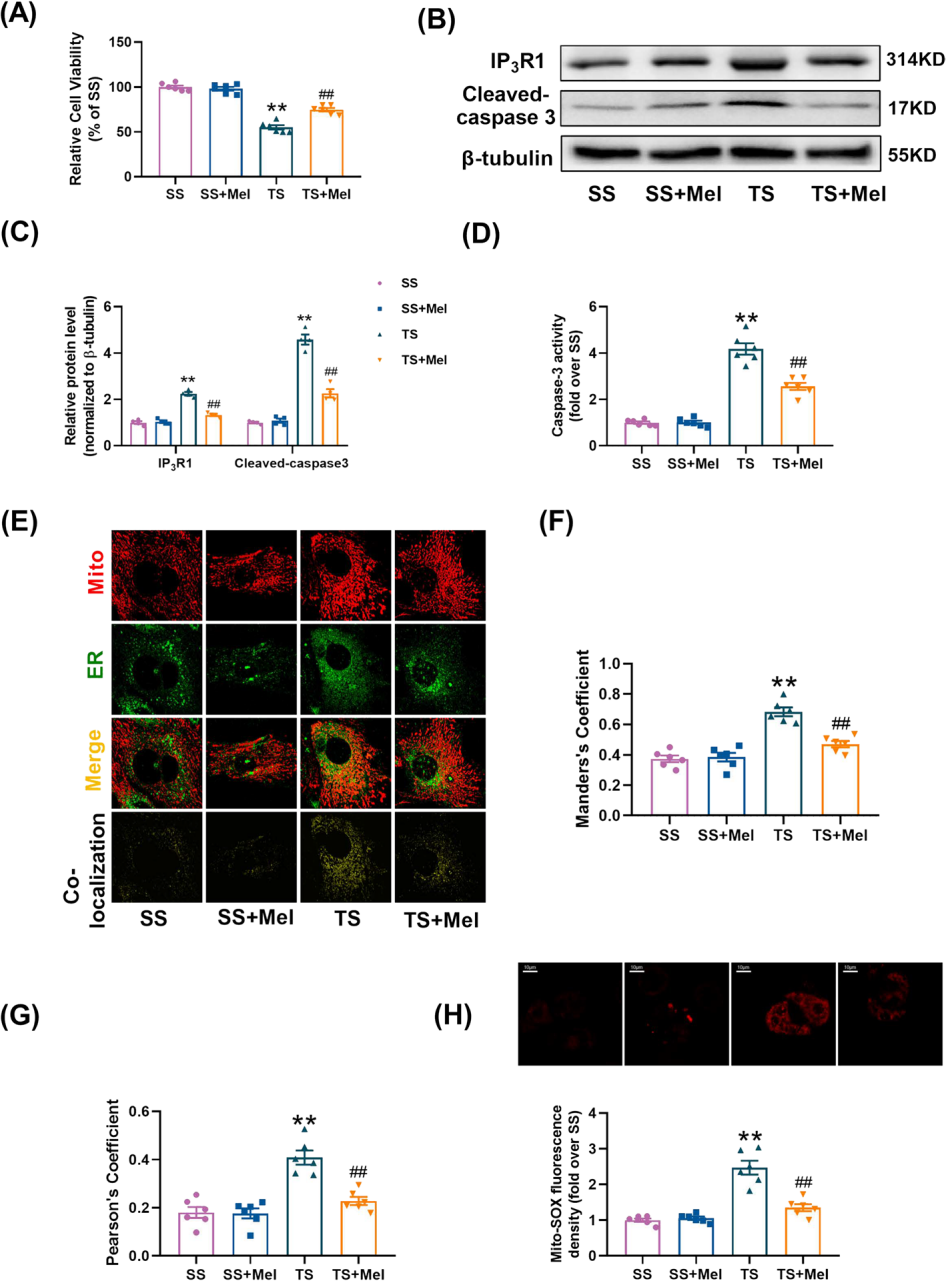
Melatonin inhibited cell death and MAMs formation in the hepatocytes cultured in TS. **A** Cell viability (percentage of SS). **B**, **C** Representative blots and quantitative analysis of IP_3_R1, cleaved-caspase 3 (*n* = 4). **D** Relative activity of caspase 3. **E** Representative confocal images of primary hepatocytes double-stained by mitotracker (red) and ER-localized virus (green) at × 600 magnification. **F**, **G** The representative figures of scatter plot were analyzed by colocalization finder in Image J software. Statistical quantification of the colocalization area between mitochondria and ER. **H** Representative images and quantitative analysis of MitoSOX-stained mitochondria-derived superoxide production. All of the values are shown as the means ± SEM. *n* = 6 in each group. **P < 0.01 vs SS; ^##^*P* < 0.01 vs TS

It was found that melatonin (100 μmol/L, 12 h) enhanced cell viability (Fig. 4A) and reduced cleaved caspase-3 expression and caspase 3 activity (Fig. 4B–D) in TS-treated cells. Under TS conditions, the expression of IP_3_R1 and the formation of MAMs were increased, as evidenced by remarkably increased Manders’s coefficient and Pearson’s coefficient between mitochondria and ER. Melatonin inhibited the elevation of IP_3_R1 and MAMs induced by TS in the hepatocytes (Fig. 4B, C,E–G). Moreover, melatonin significantly alleviated mitochondrial oxidative stress in the TS-treated hepatocytes (Fig. 4H). These results suggested that the formation of MAMs and cellular injury induced by TS were suppressed by melatonin.

### Melatonin suppressed mitochondrial calcium overload, increased mitochondrial membrane potential and improved mitochondrial function in TS-induced hepatocytes

**Fig. 5:**
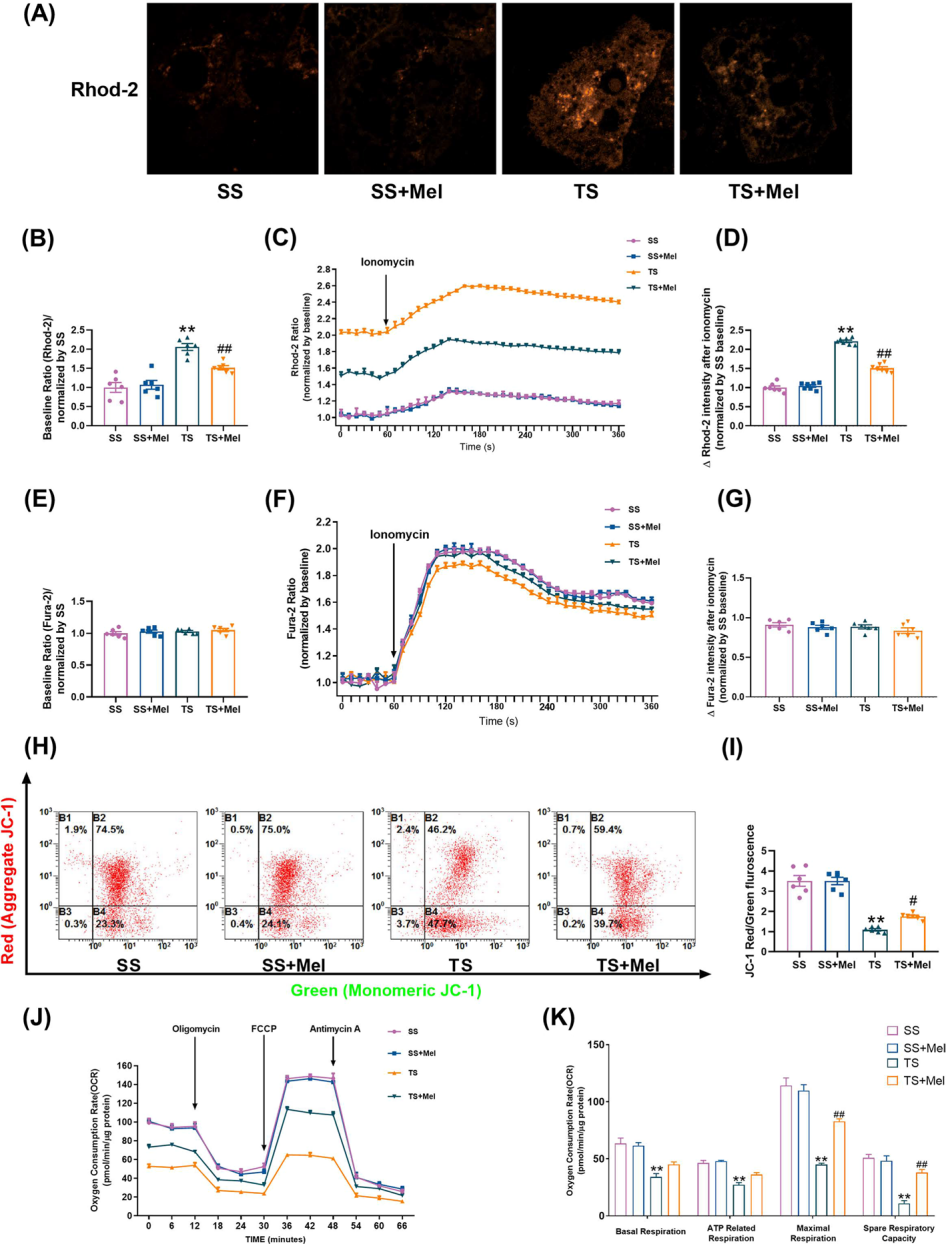
Melatonin suppressed mitochondrial calcium overload, increased mitochondrial membrane potential and improved mitochondrial function in TS-induced hepatocytes. **A**, **B** Representative confocal images of mitochondrial Ca^2+^ concentration in primary hepatocytes stained by Rhod-2 dye at × 600 magnification and quantification of fluorescence intensity normalized by SS. **C** Representative traces of ionomycin-induced changes of mitochondrial Ca^2+^ concentration (*n* = 3). **D** Quantification of the relative increment of fluorescence intensity in mitochondrial Ca^2+^ concentration after ionomycin stimulation normalized by SS. **E** Quantification of fluorescence intensity in cytosolic Ca^2+^ concentration stained by Fura-2 AM dye normalized by SS. **F** Representative traces of ionomycin-induced changes of cytosolic Ca^2+^ concentration (*n* = 3). **G** Quantification of the relative increment of fluorescence intensity in cytosolic Ca^2+^ concentration after ionomycin stimulation normalized by SS. **H**, **I** Representative flow cytometry results and statistical analysis of mitochondrial membrane potential by JC-1 stanning in primary hepatocytes. **J**, **K** Oxygen consumption rate (OCR) measured by Seahorse and quantitative statistical analysis of OCR (*n* = 3). All of the values are shown as the means ± SEM. *n* = 6 in each group. **P < 0.01 vs SS; ^##^*P* < 0.01 vs TS

The MAMs constitute a Ca^2+^ transferring channel from ER to mitochondria [44, 45]. The increase of MAMs formation in TS-induced hepatocytes here made us conjecture reasonably that abnormal mitochondrial Ca^2+^ uptake might happen. Mitochondrial Ca^2+^ concentration was detected by Rhod-2 dye. Melatonin inhibited the increase of basal mitochondrial Ca^2+^ concentration in TS-induced hepatocytes, indicating that mitochondrial Ca^2+^ overload induced by TS treatment could be alleviated by melatonin (Fig. 5A, B). Mitochondrial dynamic Ca^2+^ uptake was further detected in the stimulus of ionomycin, a selective calcium ionophore which can trigger a prompt increase in intracellular Ca^2+^ [46]. It was found that ionomycin-stimulated mitochondrial Ca^2+^ uptake in TS group was much more than that in SS group, while this alteration was inhibited by treatment of melatonin (Fig. 5C, D). Mitochondria can not only absorb Ca^2+^ from the ER through the IP_3_R1/GRP75/VDAC1 channels (MAMs), but also take up Ca^2+^ from the cytosol [47]. Fura-2 AM dye was used to determine cytosolic Ca^2+^ concentration. It was found that basal concentration (Fig. 5E, F) and increase rate (Fig. 5F, G) of Ca^2+^ were similar among different groups. These results suggested that TS-induced mitochondrial Ca^2+^ overload may predominantly come from the ER through the MAMs rather than cytosolic calcium stores.

Previous study has demonstrated that mitochondrial membrane potential (MMP) and mitochondrial function were closely related to mitochondrial Ca^2+^ concentration [47]. MMP was determined by JC-1 and the flow cytometry results showed that MMP was significantly reduced in TS-treated hepatocytes (Fig. 5H, I). The mitochondrial respiratory capacity involving basal respiration, ATP-coupled respiration, maxima respiration and spare respiration was reduced in TS-treated hepatocytes (Fig. 5J, K). Melatonin significantly reversed the loss of MMP and improved the mitochondrial respiratory capacity under TS conditions (Fig. 5H–K). These results suggested that melatonin suppressed mitochondrial calcium overload, increased mitochondrial membrane potential and improved mitochondrial function under traumatic condition.

### Melatonin inhibited MAMs formation and mitochondrial calcium overload in TS-treated hepatocytes by suppressing IP_3_R1 expression

The role of IP_3_R1 in MAMs formation and mitochondrial calcium overload was further explored. At first, siRNAs were used to knock down IP_3_R1 expression to mimic the action of melatonin. Treatment of TS resulted in a significant increase of the colocalization between ER and mitochondria, while IP_3_R1 knockdown caused a significant reduction in the colocalization, as evidenced by decreased Manders’s coefficient and Pearson’s coefficient (Additional file 1: Fig. S2A–C). Meanwhile, IP_3_R1 knockdown reduced mitochondrial oxidative stress in TS-treated hepatocytes (Additional file 1: Fig. S2D). As shown in Additional file 1: Fig. S2E–G, basal Ca^2+^ concentration and increase Ca^2+^ rate in mitochondria were inhibited by IP_3_R1 siRNA under TS condition. Next, the impacts of IP_3_R1 knockdown on apoptosis and mitochondrial function in TS-incubated cells were explored. As shown in Additional file 1: Fig. S2H–L, IP_3_R1 siRNA increased cell viability, inhibited cell apoptosis and enhanced mitochondrial respiratory capacity under TS conditions. All these results indicated that IP_3_R1 knockdown mitigated the formation of TS-induced MAMs, as well as the resultant mitochondrial calcium overload and dysfunction.

**Fig. 6:**
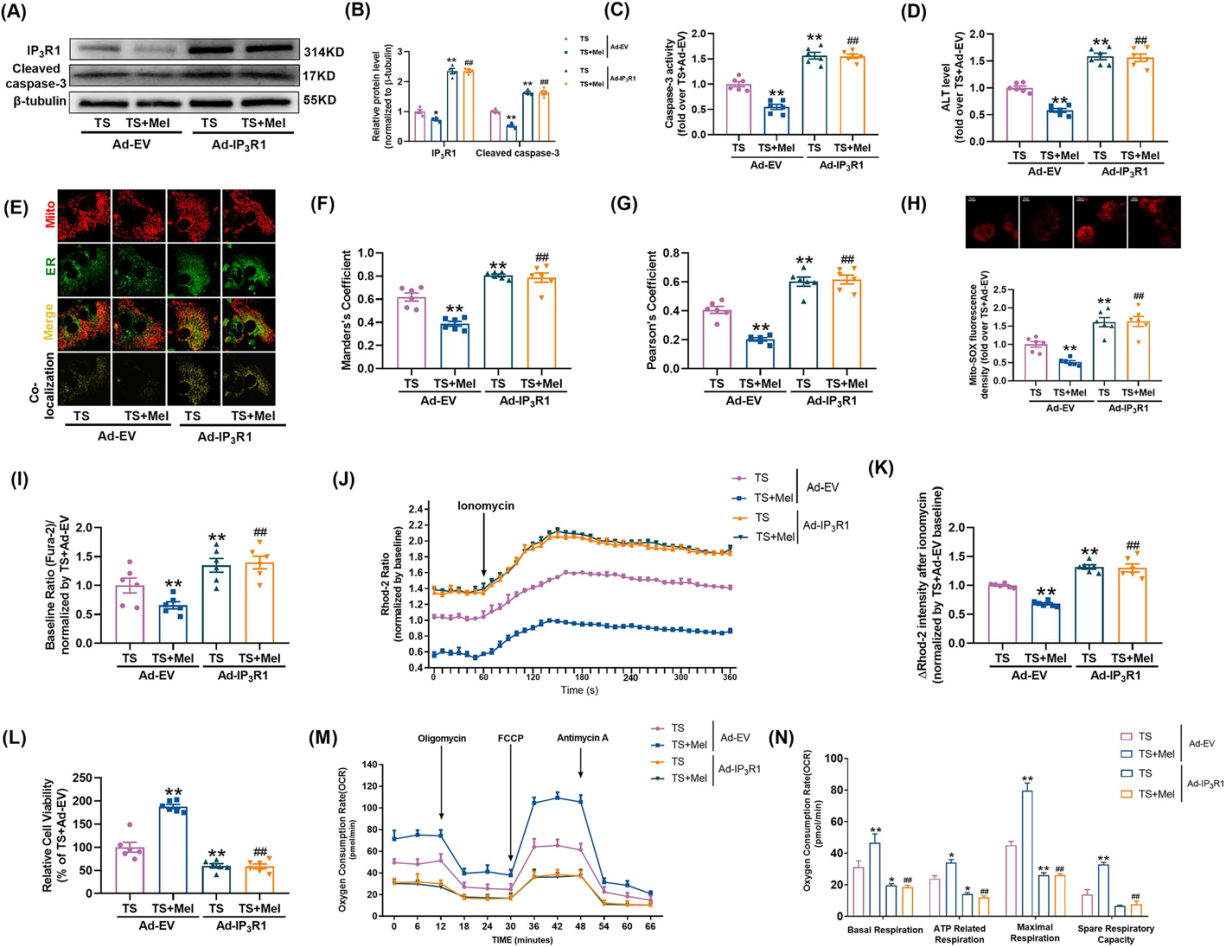
Melatonin inhibited MAMs formation and mitochondrial calcium overload in TS-treated hepatocytes by suppressing IP_3_R1 expression. **A**, **B** Representative blots and quantitative analysis of IP_3_R1, cleaved-caspase 3 (*n* = 4). **C** Relative activity of caspase 3. **D** The levels of ALT release (fold difference compared with TS + Ad-EV). **E** Representative confocal images of primary hepatocytes double-stained by mitotracker (red) and ER-localized virus (green) at × 600 magnification. **F**, **G** Statistical quantification of the colocalization area between mitochondria and ER. **H** Representative images and quantitative analysis of MitoSOX-stained mitochondria-derived superoxide production. **I** Quantification of fluorescence intensity normalized by TS + Ad-EV. **J** Representative traces of ionomycin-induced changes of mitochondrial Ca^2+^ concentration (*n* = 3). **K** Quantification of the relative increment of fluorescence intensity in mitochondrial Ca^2+^ concentration after ionomycin stimulation normalized by TS + Ad-EV. **L** Cell viability (percentage of TS + Ad-EV). **M**, **N** Oxygen consumption rate (OCR) measured by Seahorse and quantitative statistical analysis of OCR (*n* = 3). All of the values are shown as the means ± SEM. *n* = 6 in each group. *P < 0.05, **P < 0.01 vs TS + Ad-EV; ^##^*P* < 0.01 vs TS + Mel + Ad-EV

Secondly, to explore the role of IP_3_R1 in melatonin-induced hepatocellular protection, adenovirus encoding IP_3_R1 (Ad-IP_3_R1) was transfected into primary hepatocytes before treatment of melatonin. Melatonin’s inhibitory effects on apoptosis (Fig. 6A–C), ALT release (Fig. 6D), MAMs formation (Fig. 6E–G), mitochondrial oxidative stress (Fig. 6H) and mitochondrial calcium overload (Fig. 6I–K) were blunted in the TS-treated primary hepatocytes when IP_3_R1 was overexpressed. Meanwhile, overexpression of IP_3_R1 weakened the effect of melatonin to improve cell viability (Fig. 6L) and mitochondrial oxidative phosphorylation capacity (Fig. 6M, N) in the TS-treated primary hepatocytes. These results indicated that melatonin inhibited MAMs formation and mitochondrial calcium overload and alleviated hepatocellular injury under traumatic condition via suppression of IP_3_R1.

### Melatonin inhibited IP_3_R1-mediated MAMs via ERK1/2 signaling pathway

**Fig. 7:**
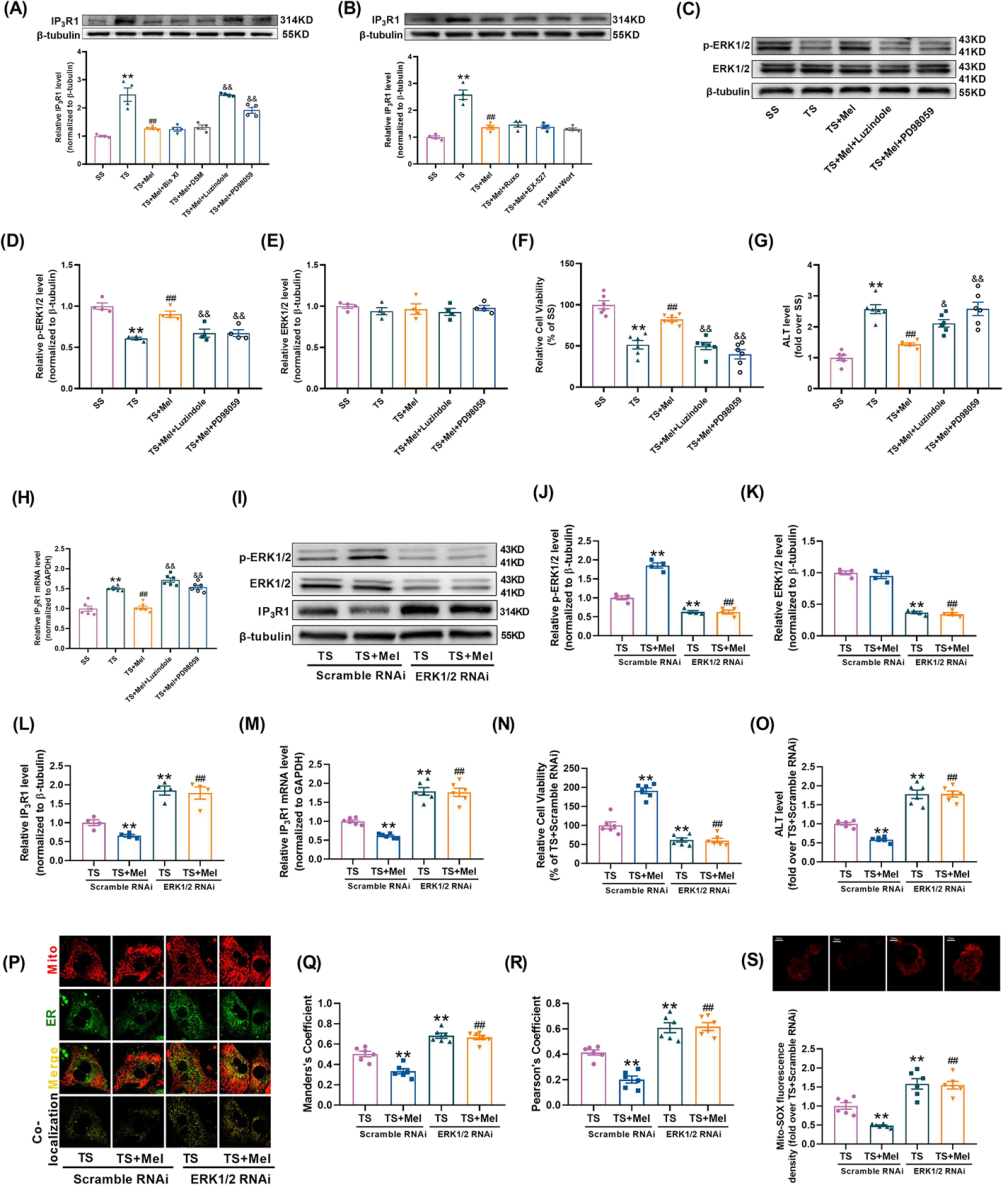
Melatonin inhibited IP_3_R1-mediated MAMs via ERK1/2 signaling pathway. **A**, **B** Several specific pharmacological inhibitors, including Bisindolylmaleimide XI hydrochloride (Bis XI, a PKC inhibitor), Dorsomorphin (DSM, an AMPK inhibitor), PD98059 (a MEK/ERK inhibitor), Luzindole (a melatonin receptor inhibitor), Ruxolitinib (Ruxo, a JAK inhibitor), EX-527 (a SIRT1 inhibitor) and Wortmannin (a PI3K/Akt inhibitor) were pre-administered to the hepatocytes in the TS + Mel group and then the expression of IP_3_R1 was quantified (*n* = 4). **C**, **E** Representative blots and quantitative analysis of phosphorylated ERK1/2 (p-ERK1/2) and total ERK1/2 (*n* = 4). **F** Cell viability (percentage of SS). **G** The levels of ALT release (fold difference compared with SS). **H** Quantitative analysis of IP_3_R1 mRNA expression determined by real-time PCR. **I–L** ERK1/2 was knocked down by siRNA, after which the cells were subjected to TS with or without melatonin. Representative blots and quantitative analysis of phosphorylated ERK1/2 (p-ERK1/2), total ERK1/2 and IP_3_R1 (*n* = 4). **M** Quantitative analysis of IP_3_R1 mRNA expression determined by real-time PCR. **N** Cell viability (percentage of TS + Scramble RNAi). **O** The levels of ALT release (fold difference compared with TS + Scramble RNAi). **P** Representative confocal images of primary hepatocytes double-stained by mitotracker (red) and ER-tracker (green) at × 600 magnification. **Q**, **R** Statistical quantification of the colocalization area between mitochondria and ER. **S** Representative images and quantitative analysis of MitoSOX-stained mitochondria-derived superoxide production. All of the values are shown as the means ± SEM. *n* = 6 in each group. *P < 0.05, **P < 0.01 vs SS or TS + Scramble RNAi; ^##^*P* < 0.01 vs TS or TS + Mel + Scramble RNAi; ^&^*P* < 0.05 ^&&^*P* < 0.01 vs TS + Mel

To further clarify the signaling pathway mediating the downregulation of IP_3_R1 by melatonin, melatonin receptor inhibitor and several inhibitors of intracellular melatonin signaling pathways were utilized to hinder the possible signaling pathway [48–50]. The hepatocytes in the TS + Mel group were cultured with the following inhibitors: Bisindolylmaleimide XI hydrochloride (Bis XI, a PKC inhibitor targeting isoform, 2 µM; MedChem Express) [51], Dorsomorphin (DSM, a AMPK inhibitor, 2 µM; MedChem Express) [52], Luzindole (a melatonin receptor inhibitor, 1 µM; MedChem Express) [53], PD98059 (a MEK/ERK inhibitor, 10 µM; MedChem Express) [54], Ruxolitinib (Ruxo, a JAK inhibitor, 1 µM; MedChem Express) [55], EX-527 (a SIRT1 inhibitor, 1 µM; MedChem Express) [56] and Wortmannin (a PI3K/Akt inhibitor, 0.1 µM; MedChem Express) [57]. In the TS-treated hepatocytes, pretreatment with Luzindole or PD98059 significantly abrogated melatonin-induced reduction of IP_3_R1, while other inhibitors had no significant effects (Fig. 7A, B). Subsequently, we observed that melatonin-induced increase of p-ERK1/2 expression was blunted by Luzindole or PD98059 (Fig. 7C–E), which suggested that melatonin receptor (MR) was an upstream regulator of ERK1/2 activation. Moreover, pretreatment with Luzindole or PD98059 not only blocked melatonin’s promoting effects on cell viability (Fig. 7F), but also blunted melatonin’s inhibition on ALT release (Fig. 7G) and IP_3_R1 mRNA level (Fig. 7H) in the TS-treated hepatocytes.

ERK1/2 siRNA was further utilized to ascertain whether ERK1/2 was accountable for melatonin’s inhibition of IP_3_R1-mediated MAMs formation. As shown in Fig. 7I–M, knockdown of ERK1/2 reduced the expression of ERK1/2 and enhanced IP_3_R1 expression in the TS-treated hepatocytes. In addition, knockdown of ERK1/2 not only blunted the promoting effects of melatonin on cell viability (Fig. 7N), but also weakened the inhibitory effects of melatonin on ALT release, IP_3_R1-mediated MAMs formation and mitochondrial oxidative stress (Fig. 7O–S) in the TS-treated hepatocytes. These data indicated melatonin's suppressive effects on IP_3_R1-mediated MAMs formation were largely attributed to the activation of ERK1/2.

### Melatonin-ERK1/2 suppressed IP_3_R1-mediated MAMs via inhibiting FoxO1-mediated transcription

**Fig. 8:**
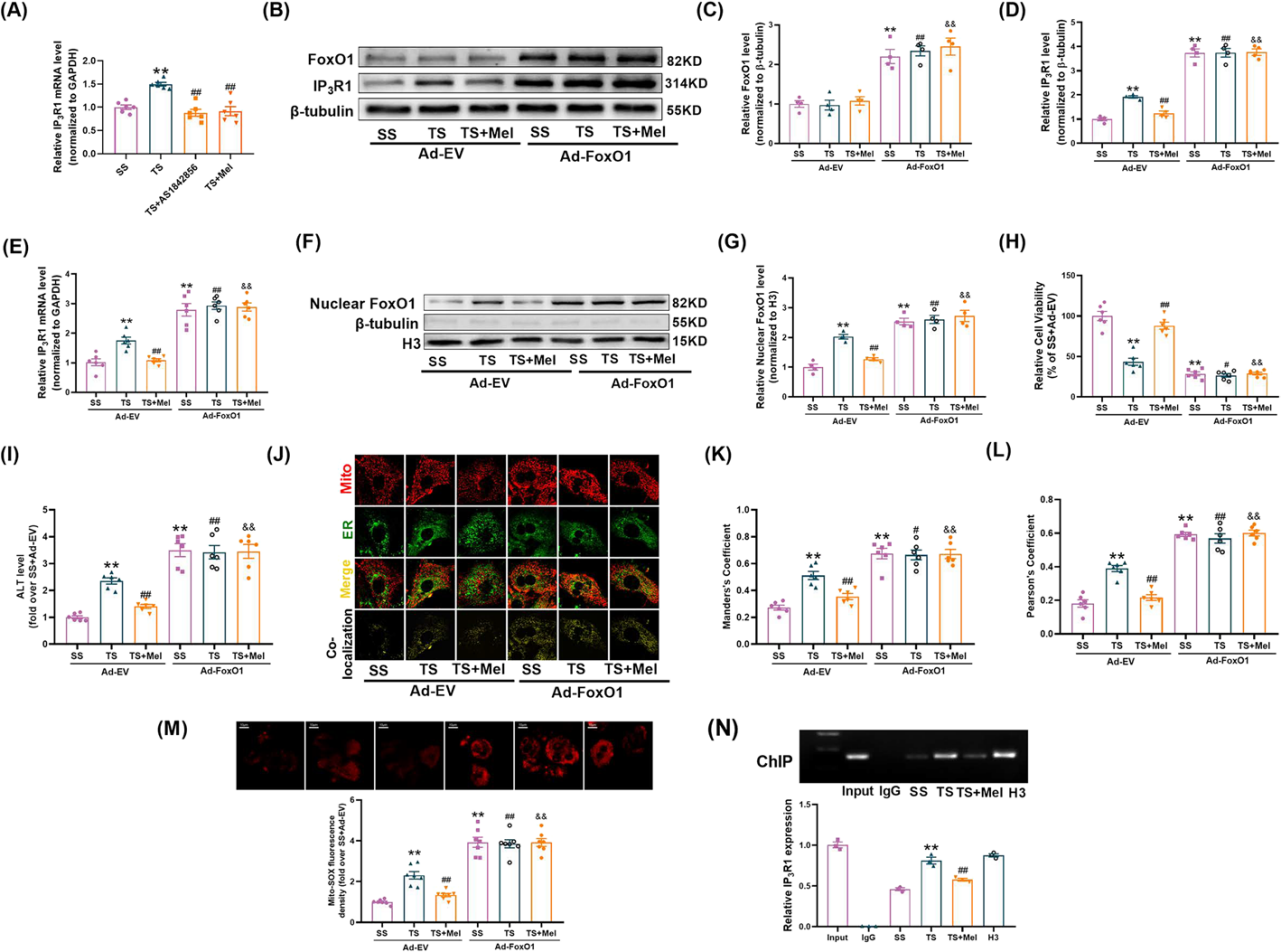
Melatonin-ERK1/2 suppressed IP_3_R1-mediated MAMs via inhibiting FoxO1-mediated transcription. **A** Quantitative analysis of IP_3_R1 mRNA expression determined by real-time PCR (AS1842856, a JUNB inhibitor) (*n* = 6). **B–D** FoxO1 was overexpressed by adenovirus, after which the cells were subjected to TS or TS + Mel. Representative blots and quantitative analysis of FoxO1 and IP_3_R1 (*n* = 4). **E** Quantitative analysis of IP_3_R1 mRNA expression determined by real-time PCR. **F**, **G** Representative blots and quantitative analysis of nuclear FoxO1 (*n* = 4). **H** Cell viability (percentage of SS + Ad-EV). **I** The levels of ALT release (fold difference compared with SS + Ad-EV). **J** Representative confocal images of primary hepatocytes double-stained by mitotracker (red) and ER-localized virus (green) at × 600 magnification. **K**, **L** Statistical quantification of the colocalization area between mitochondria and ER. **M** Representative images and quantitative analysis of MitoSOX-stained mitochondria-derived superoxide production. **N** Chromatin immunoprecipitation (ChIP) and real-time PCR analysis for the binding of FoxO1 to IP_3_R1 promoter (*n* = 3). All of the values are shown as the means ± SEM. *n* = 6 in each group. **P < 0.01 vs SS or SS + Ad-EV; ^#^*P* < 0.05, ^##^*P* < 0.01 vs TS + Ad-EV; ^&&^*P* < 0.01 vs TS + Mel + Ad-EV

Given that IP_3_R1 was regulated on the mRNA level (Fig. 7M) and ERK1/2 is a mitogen-activated protein kinase, potential transcription factors (TFs) that can be regulated by phosphorylation and interact with IP_3_R1 promoter region were screened. Among them, five TFs were known to be regulated by phosphorylation, including FoxO1, JUNB, MEF2A, MEF2C, Nfatc2. To determine whether the five candidate TFs transcriptionally regulated IP_3_R1, we constructed luciferase reporter plasmids containing IP_3_R1-promoter sequence. Luciferase reporter assays revealed that FoxO1 or JUNB significantly increased IP_3_R1 promoter activity, while the other TFs had no significant effects on IP_3_R1 promoter activity (Additional file 1: Fig. S3A). The effects of TS or melatonin on nuclear expressions of FoxO1 and JUNB (involved in regulating transcription) were then explored. As shown in Additional file 1: Fig. S3B-D, TS increased the nuclear expression of FoxO1 but not JUNB. Melatonin reduced the nuclear expression of FoxO1 in the TS-treated hepatocytes, which was blunted by the ERK specific inhibitor PD98059. Knockdown of ERK1/2 reduced the phosphorylation of FoxO1 (Additional file 1: Fig. S3E-G) and the expression of nuclear FoxO1 (Additional file 1: Fig. S3H) in the TS-treated hepatocytes. Co-IP assays showed that ERK1/2 co-precipitated with endogenous FoxO1. Treatment with TS reduced the binding of ERK1/2 and FoxO1, which was improved by treatment of melatonin (Additional file 1: Fig. S3I). The animal experimental study validated that melatonin increased ERK1/2 and FoxO1 phosphorylation and reduced level of nuclear FoxO1 in the MT-treated livers (Additional file 1: Fig. S4A-G). Moreover, the mRNA expression of IP_3_R1 was inhibited by FoxO1 inhibitor (AS1842856) or melatonin under TS condition (Fig. 8A). These results indicated that FoxO1 may be the molecular link between ERK1/2 and IP_3_R1 signaling.

To further verify whether decreased FoxO1 was necessary for controlling IP_3_R1-mediated MAMs formation in response to treatment of melatonin, FoxO1 was overexpressed by using adenovirus. It was shown that overexpression of FoxO1 not only increased the expression of total FoxO1, nuclear FoxO1, IP_3_R1 (Fig. 8B–G), ALT release (Fig. 8I), IP_3_R1-mediated MAMs formation and mitochondrial oxidative stress (Fig. 8J–M), but also inhibited cell viability under SS conditions (Fig. 8H). Meanwhile, overexpression of FoxO1 hindered the protection of melatonin in the TS-treated hepatocytes (Fig. 8B–M). Chromatin immunoprecipitation (ChIP)-PCR assays indicated that FoxO1 was directly bound to the promoter of IP_3_R1. Treatment with TS increased the binding of FoxO1 to the promoter of IP_3_R1, which was inhibited by administration of melatonin (Fig. 8N). Taken together, these results suggested that FoxO1 was directly responsible for increased IP_3_R1-mediated MAMs formation and decreased cell viability in traumatic hepatocytes.

## Discussion

**Fig. 9:**
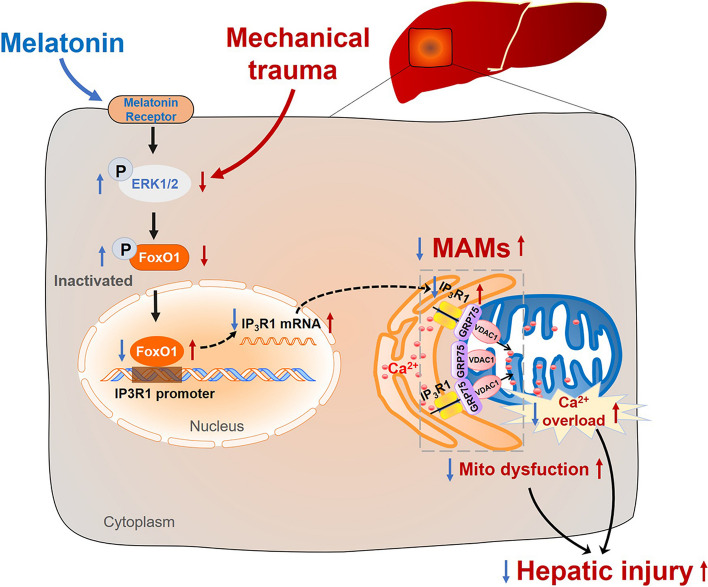
Schematic figure showing that melatonin attenuates mechanical trauma-induced hepatic injury by inhibiting IP_3_R1-mediated MAMs formation via activating the melatonin receptor-ERK1/2-FoxO1 pathway. Melatonin binds to melatonin receptor and induces the phosphorylation of ERK1/2 that subsequently interacts with FoxO1 and induces the phosphorylation and inactivation of the FoxO1. Inactivated FoxO1 reduced FoxO1 binding to the IP_3_R1 promoter to inhibit IP_3_R1-mediated MAMs. Subsequently, melatonin-induced downregulation of IP_3_R1 inhibits MAMs formation and Ca^2+^ overload, and mitigates mitochondrial dysfunction and mechanical trauma-induced hepatic injury

Our study presented for the first time that melatonin is identified as a regulator of MAMs to preserve mitochondrial function and protect against mechanical trauma-induced hepatic injury. Mechanistically, using inhibitor screening and gain- or loss-of-function analysis, it is demonstrated that melatonin activates ERK1/2 and ERK1/2 subsequently interacts with FoxO1 and induces FoxO1 inactivation, which reduces the binding of FoxO1 to the promoter of IP_3_R1 and subsequently inhibits IP_3_R1-mediated MAMs formation (Fig. 9). In conclusion, melatonin is identified to be a promising therapeutic agent against MT-induced secondary liver damages and elaborates the detailed molecular mechanism on how melatonin inhibits IP_3_R1-mediated MAMs formation to exert hepatic protective effects.

The physical and functional connection between the ER and mitochondria occurs through MAMs. This connection ensures efficient and dynamic transmission of physiological as well as pathological Ca^2+^ signals between the two organelles [58]. ER and mitochondria can interact in the range of 10–20 nm [59]. Many of the proteins in mitochondria-ER structural coupling are related to Ca^2+^, such as IP_3_R, GRP75, Mfn2, Ca^2+^-binding chaperonin and so on, which form an effective mechanism of Ca^2+^ transport between mitochondria and ER [60]. Research has indicated that when IP_3_Rs channels are opened, Ca^2+^ flows out of the ER, causing an enhanced local Ca^2+^ concentration in MAMs, and then Ca^2+^ can enter into mitochondria. On the other hand, VDAC1 is structurally coupled with IP_3_Rs via GRP75 to promote Ca^2+^ exchange between the ER and mitochondria [12]. In our present study, there was an increase in the formation of IP_3_R1-mediated MAMs in the cells cultured with TS, which was accompanied by accumulation of mitochondrial calcium, the reduction in mitochondrial membrane potential (∆Ψm), and the decline in mitochondrial oxidative phosphorylation capacity. Treatment of melatonin inhibited IP_3_R1-mediated MAMs, alleviated mitochondrial calcium overload and improved mitochondrial function. These results supported the conclusion that abnormal expression of proteins in the MAMs contributes to mitochondrial calcium overload and mitochondrial dysfunction during MT-induced hepatocyte injury, which expands the understanding of previous studies [21] about the role of disturbed MAMs in hepatic diseases.

Melatonin, a lipophilic hormone synthesized and secreted mainly in the pineal gland, is considered as a potential therapeutic agent that modulates a variety of physiological activities, including the regulation of circadian rhythms, immune responses, the oxidative process, apoptosis or mitochondrial homeostasis via receptor-dependent (membrane, cytosolic, and nuclear receptors) or receptor-independent mechanisms [61, 62]. Three types of receptor-mediated pathways are activated: membrane receptors, including MR1 and MR2, cytosolic receptor (MR3) and nuclear receptors. It has been demonstrated that both MR1 and MR2 are highly homologous and bind melatonin with a high affinity. Activation of MR1 and MR2 triggers separation of the G-protein, G-α subunit, and G-βγ complex heterotrimers, thus triggering effector proteins in the downstream pathway [63] including ERK1/2 [64], JNK [65]. The MR3 receptor is primarily involved in detoxification as an enzyme called quinone oxidoreductase and exhibits low affinity for melatonin in the cytosol. The third nuclear receptors-retinoid orphan receptors (ROR)-dependent pathway may produce antioxidants and an anti-inflammatory effect [66, 67]. In addition, melatonin can directly interact with other molecules and exert its antioxidant and radical scavenging actions [61]. In our results, melatonin inhibited IP_3_R1 expression through MR1 and MR2-dependent pathway, which suggested that the inhibition of IP_3_R1-mediated MAMs is not a direct anti-oxidant effect of melatonin.

Through several experimental screening, our study has found that Foxo1 is the linking molecule between ERK1/2 and IP_3_R1. ERK1/2 activation is generally associated with an increase in inflammation and the synthesis of pro-inflammatory cytokines [68, 69], while one study has shown that phosphorylated ERK1/2 expression is down-regulated in hepatectomy-induced acute liver failure with extensive hepatocyte apoptosis [70]. The exact mechanism of reduced ERK1/2 phosphorylation after trauma is currently unclear in our study. The reason for reduced ERK1/2 phosphorylation of liver in the trauma group compared to the sham group may be caused by factors other than inflammation in the animal model. After all, the changes in the liver under trauma are very complex, and the reasons remain to be elucidated by further studies. As a transcription factor, the phosphorylation of FoxO1 by kinase at Ser 256 results in the export of FoxO1 from the nucleus to the cytoplasm, thereby inhibiting its transcriptional activity. Asada et al. [71] have shown that ERK and p38 of the MAPK family directly phosphorylate Foxo1 at Ser 256 in vitro or in vivo. Consistent with previous studies, our study has found that melatonin promotes the phosphorylation and inhibits the activation of FoxO1 at Ser 256 through the phosphorylation of ERK1/2. FoxO1 has been shown to transcriptionally regulate key gluconeogenesis enzymes such as G6Pase and PEPCK [72]. Our recent study showed that FoxO1 downregulates transcription of mitochondrial fusion protein Mfn2 [73]. One important finding in our study is that we have revealed a novel transcriptional regulation of FoxO1 on IP_3_R1-mediated MAMs formation. It is demonstrated that FoxO1 directly binds IP_3_R1 promoter, and TS treatment increased FoxO1 binding to the IP_3_R1 promoter, which was inhibited by treatment of melatonin. These findings demonstrate that IP_3_R1 is a new transcriptional target of FoxO1.

There are still some limitations in our study. First, we explored the role ERK1/2-FoxO1-IP_3_R1 signaling in the protective effects of melatonin primarily at the cellular level using ERK1/2 siRNA and FoxO1 or IP_3_R1 adenovirus, and the use of hepatic specific knockout or overexpression animals will be very useful in further elucidating the role of ERK1/2-FoxO1 in IP_3_R1-mediated MAMs formation. Second, reduced phosphorylation of hepatic ERK1/2 after trauma may be caused by factors other than inflammation, and the exact mechanism is currently unclear. Despite these limitations, our study has demonstrated a critical role of IP_3_R1-mediated MAMs formation in MT-induced hepatic injury. Melatonin prevented the MAMs formation via MR-ERK1/2-FoxO1-IP_3_R1 and exerted protective effects against post-traumatic hepatic dysfunction.

Taken together, our study has provided novel insights into the pathogenesis of post-traumatic hepatic injury, high-lighting that melatonin may be a useful therapeutic candidate for MT patients to prevent secondary hepatic injury. In addition, MR-ERK1/2-FoxO1-IP_3_R1 signaling pathway interventions also are a potential therapeutic strategy. The characteristics of melatonin in preserving the balance of MAMs dynamics bring a new path to reveal its broad hepatic protect effects.

## Conclusion

Our study indicates that mechanical trauma causes hepatic injury by triggering IP_3_R1-mediated MAMs formation. Melatonin prevents MAMs formation and alleviates hepatic injury through ERK1/2-FoxO1 pathway, which negatively regulates the expression of IP_3_R1 by decreasing of FoxO1 binding to the IP_3_R1 promoter. These findings suggest that melatonin-modulated MAMs could be used to treat MT-induced hepatic injury.

### Supplementary Information

**Additional file 1:** **Figure** **S1** Mediators that cause mechanical trauma-induced hepatic injury mainly existed in the serum of MT rats. **(A)** Schematic representation of the experimental grouping. (B-C) Determination of hepatocytes apoptosis by flow cytometry with Annexin V and PI staining and the apoptotic statistical graph. NH: normal hepatocytes; TH: traumatic hepatocytes; SS: 20% diluted serum in Sham rats; TS: 20% diluted serum in MT (after 4 hour) rats. (D) Cell viability in the hepatocytes incubated with a series of diluted SS and TS. (E) Serum IL-1β level of MT rats. *n*=8 (F) Serum IL-6 level of MT rats. *n*=8. (G) Serum TGFα level of MT rats. *n*=8 (H) Cell viability (percentage of SS). (I) Serum IL-1β level. All of the values are shown as the means ± SEM. *n*=6 in each group. **P < 0.01 vs NH + SS, Ctrl, Sham, or SS. *P < 0.05 vs Sham. ^##^*P* < 0.01 vs TH + SS or MT+V. ^#^*P* < 0.05 vs MT+V. **Figure** **S2** Knockdown of IP_3_R1 alleviated TS-induced MAM formation and mitochondrial calcium overload and mitochondrial dysfunction. (A) Representative confocal images of primary hepatocytes double-stained by mitotracker (red) and ER-tracker (green) at ×600 magnification. (B-C) Statistical quantification of the colocalization area between mitochondria and ER. (D) Representative images and quantitative analysis of MitoSOX-stained mitochondria-derived superoxide production. (E) Quantification of fluorescence intensity normalized by SS + Scramble RNAi. (F) Representative traces of ionomycin-induced changes of mitochondrial Ca^2+^ concentration (*n*=3). (G) Quantification of the relative increment of fluorescence intensity in mitochondrial Ca^2+^ concentration after ionomycin stimulation normalized by SS + Scramble RNAi. (H-I) Representative flow cytometry results and statistical analysis of mitochondrial membrane potential by JC-1 stanning in primary hepatocytes. (J) Cell viability (percentage of SS + Scramble RNAi). (K-L) Oxygen consumption rate (OCR) measured by Seahorse and quantitative statistical analysis of OCR (*n*=3). All of the values are shown as the means ± SEM. *n*=6 in each group. ^**^P< 0.01 vs SS + Scramble RNAi; ^#^*P*< 0.05, ^##^*P* < 0.01 vs TS + Scramble RNAi. **Figure** **S3** Transcription factor FoxO1 rather than JUNB suppressed IP_3_R1-mediated MAMs. (A) FoxO1 and JUNB inhibited the luciferase activity of the IP_3_R1 promoter (*n*=3). (B-D) Representative blots and quantitative analysis of nuclear FoxO1 and JUNB (PD98059, a MEK/ERK inhibitor). (E-H) ERK1/2 was knocked down by siRNA, after which the cells were subjected to TS with or without melatonin. Representative blots and quantitative analysis of phosphorylated FoxO1 (p- FoxO1), total ERK1/2 and nuclear FoxO1. (I) Interaction between ERK1/2 and FoxO1 determined by co-immunoprecipitation (*n*=3). All of the values are shown as the means ± SEM. *n*=4 in each group. ^**^P< 0.01 vs Ctrl or SS or TS + Scramble RNAi; ^##^*P* < 0.01 vs TS or TS + Scramble RNAi; ^&&^*P* < 0.01 vs TS + Mel. **Figure** **S4** Mel increased ERK1/2 and FoxO1 phosphorylation and expression of nuclear FoxO1 in MT-treated livers in vivo. (A-G) Representative blots and quantitative analysis of phosphorylated ERK1/2 (p-ERK1/2), ERK1/2, phosphorylated FoxO1 (p-FoxO1), FoxO1, and nuclear FoxO1; Mel, melatonin at a dosage of 30 mg/kg. *n* = 4 in each group. All data are shown as means ± SEM. ^**^P< 0.01 vs Sham; ^##^*P*< 0.01 vs MT + V.

**Additional** **file** **2:** **Table** **S1.** Primer sequences used in the study. **Table** **S2.** The antibodies used for western blotting (WB), Co-immunoprecipitation (Co-IP) and Chromatin immunoprecipitation (ChIP).

## Data Availability

The datasets used and/or analyzed during the current study are available from the corresponding author on reasonable request.

## Abbreviations

MAMs: Mitochondria-associated ER membranes
MT: Mechanical trauma
MR: Melatonin receptor
SS: Sham serum
TS: Trauma serum
ER: Endoplasmic reticulum
IP_3_R: Inositol 1,4,5-trisphosphate receptor
VDAC1: Voltage-dependent anion channel 1
GRP75: Glucose-regulated protein 75
Mfn2: Mitofusin 2
Bap31: B-cell receptor-associated protein 31
Fis1: Mitochondrial fission 1 protein
ALT: Alanine aminotransferase
AST: Aspartate aminotransferase
MMP: Mitochondrial membrane potential
OCR: Oxygen consumption rate
ChIP: Chromatin immunoprecipitation
Co-IP: Co-immunoprecipitation
