# Supplementary material for: IP3R1-mediated MAMs formation contributes to mechanical trauma-induced hepatic injury and the protective effect of melatonin
Source: Cell Mol Biol Lett. 2024 Feb 2;29:22. doi: 10.1186/s11658-023-00509-x (PMC10836028; doi:10.1186/s11658-023-00509-x)
Supplement: Supplementary file 1 — Additional file 1: Figure S1 Mediators that cause mechanical trauma-induced hepatic injury mainly existed in the serum of MT rats. (A) Schematic representation of the experimental grouping. (B-C) Determination of hepatocytes apoptosis by flow cytometry with Annexin V and PI staining and the apoptotic statistical graph. NH: normal hepatocytes; TH: traumatic hepatocytes; SS: 20% diluted serum in Sham rats; TS: 20% diluted serum in MT (after 4 hour) rats. (D) Cell viability in the hepatocytes incubated with a series of diluted SS and TS. (E) Serum IL-1β level of MT rats. n=8 (F) Serum IL-6 level of MT rats. n=8. (G) Serum TGFα level of MT rats. n=8 (H) Cell viability (percentage of SS). (I) Serum IL-1β level. All of the values are shown as the means ± SEM. n=6 in each group. **P < 0.01 vs NH + SS, Ctrl, Sham, or SS. *P < 0.05 vs Sham. ##P < 0.01 vs TH + SS or MT+V. #P < 0.05 vs MT+V. Figure S2 Knockdown of IP3R1 alleviated TS-induced MAM formation and mitochondrial calcium overload and mitochondrial dysfunction. (A) Representative confocal images of primary hepatocytes double-stained by mitotracker (red) and ER-tracker (green) at ×600 magnification. (B-C) Statistical quantification of the colocalization area between mitochondria and ER. (D) Representative images and quantitative analysis of MitoSOX-stained mitochondria-derived superoxide production. (E) Quantification of fluorescence intensity normalized by SS + Scramble RNAi. (F) Representative traces of ionomycin-induced changes of mitochondrial Ca2+ concentration (n=3). (G) Quantification of the relative increment of fluorescence intensity in mitochondrial Ca2+ concentration after ionomycin stimulation normalized by SS + Scramble RNAi. (H-I) Representative flow cytometry results and statistical analysis of mitochondrial membrane potential by JC-1 stanning in primary hepatocytes. (J) Cell viability (percentage of SS + Scramble RNAi). (K-L) Oxygen consumption rate (OCR) measured by Seahorse and quantitative statistica [file 11658_2023_509_MOESM1_ESM.docx]

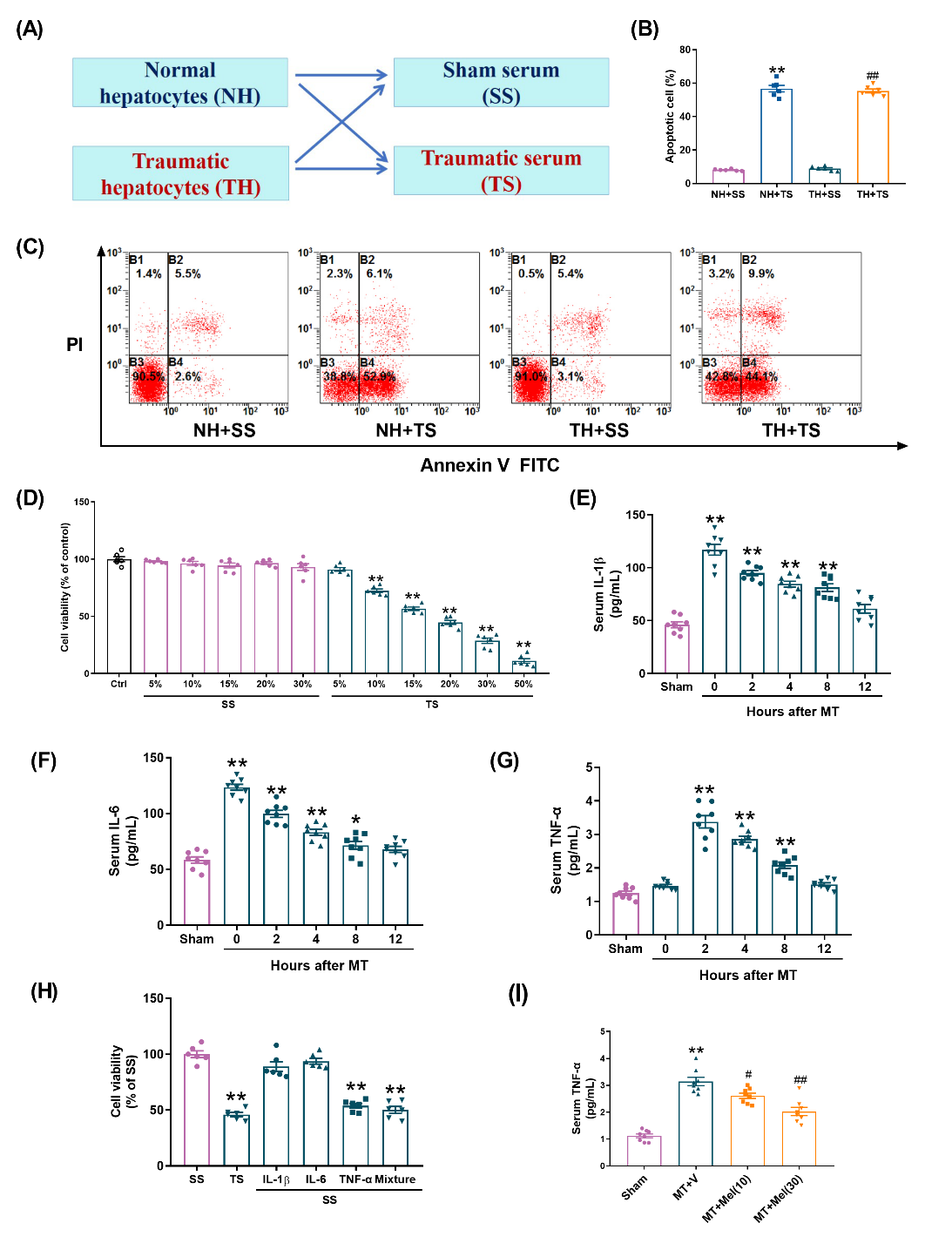
**Additional Figures and Figure Legends**

**Figure S1** Mediators that cause mechanical trauma-induced hepatic injury mainly existed in the serum of MT rats. **(A)** Schematic representation of the experimental grouping. **(B-C)** Determination of hepatocytes apoptosis by flow cytometry with Annexin V and PI staining and the apoptotic statistical graph. NH: normal hepatocytes; TH: traumatic hepatocytes; SS: 20% diluted serum in Sham rats; TS: 20% diluted serum in MT (after 4 hour) rats. **(D)** Cell viability in the hepatocytes incubated with a series of diluted SS and TS. **(E)** Serum IL-1β level of MT rats. n=8 **(F)** Serum IL-6 level of MT rats. n=8. **(G)** Serum TGFα level of MT rats. n=8 **(H)** Cell viability (percentage of SS). **(I)** Serum IL-1β level. All of the values are shown as the means ± SEM. n=6 in each group. ***P* < 0.01 *vs* NH + SS, Ctrl, Sham, or SS. **P* < 0.05 *vs* Sham. ^##^*P* < 0.01 *vs* TH + SS or MT+V. ^#^*P* < 0.05 *vs* MT+V.


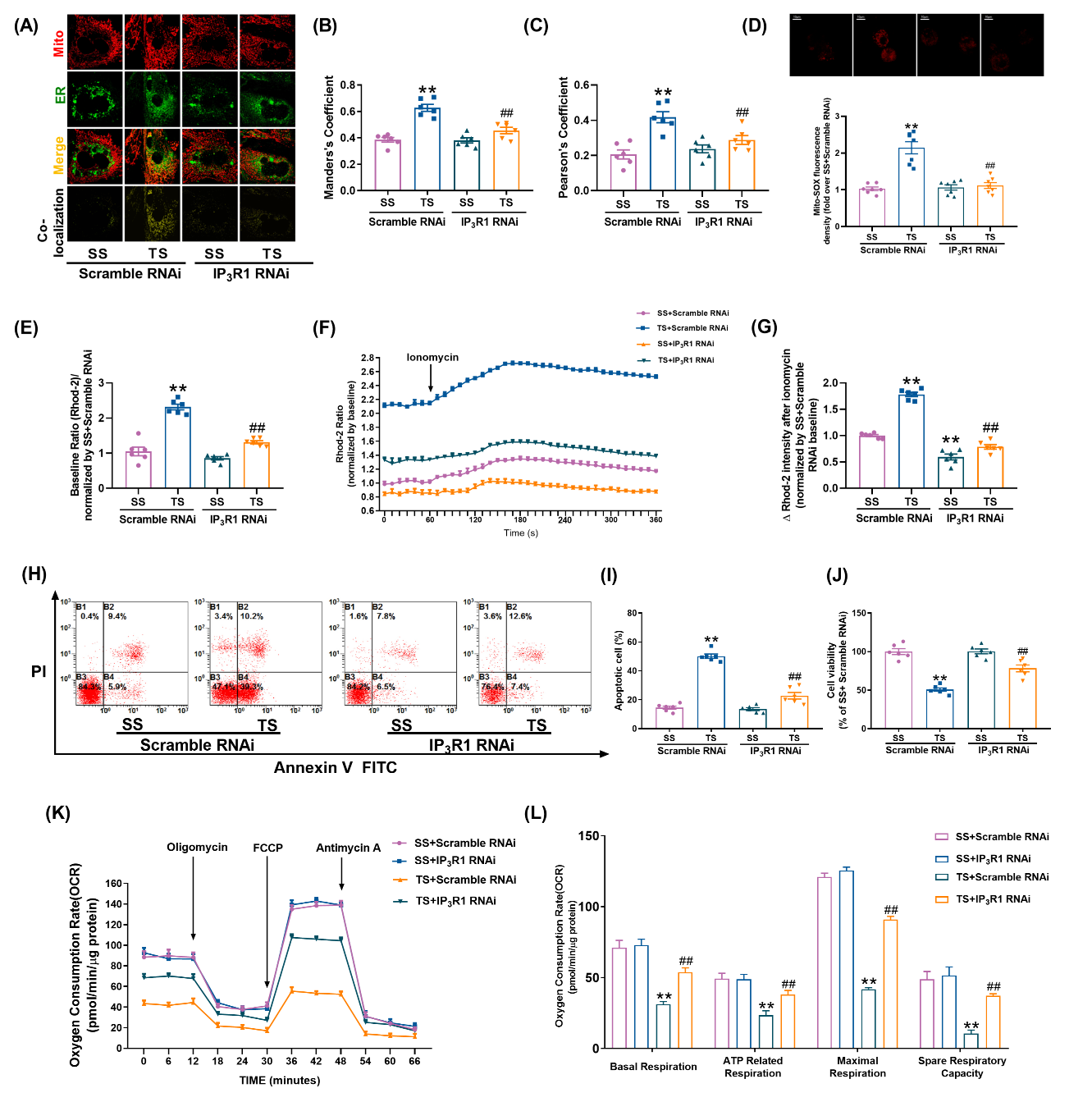
**Figure S2** Knockdown of IP_3_R1 alleviated TS-induced MAM formation and mitochondrial calcium overload and mitochondrial dysfunction. **(A)** Representative confocal images of primary hepatocytes double-stained by mitotracker (red) and ER-tracker (green) at ×600 magnification. **(B-C)** Statistical quantification of the colocalization area between mitochondria and ER. **(D)** Representative images and quantitative analysis of MitoSOX-stained mitochondria-derived superoxide production. **(E)** Quantification of fluorescence intensity normalized by SS + Scramble RNAi. **(F)** Representative traces of ionomycin-induced changes of mitochondrial Ca^2+^ concentration (n=3). **(G)** Quantification of the relative increment of fluorescence intensity in mitochondrial Ca^2+^ concentration after ionomycin stimulation normalized by SS + Scramble RNAi. **(H-I)** Representative flow cytometry results and statistical analysis of mitochondrial membrane potential by JC-1 stanning in primary hepatocytes. **(J)** Cell viability (percentage of SS + Scramble RNAi). **(K-L)** Oxygen consumption rate (OCR) measured by Seahorse and quantitative statistical analysis of OCR (n=3). All of the values are shown as the means ± SEM. n=6 in each group. ^**^*P*< 0.01 *vs* SS + Scramble RNAi; ^#^*P*< 0.05, ^##^*P* < 0.01 *vs* TS + Scramble RNAi.


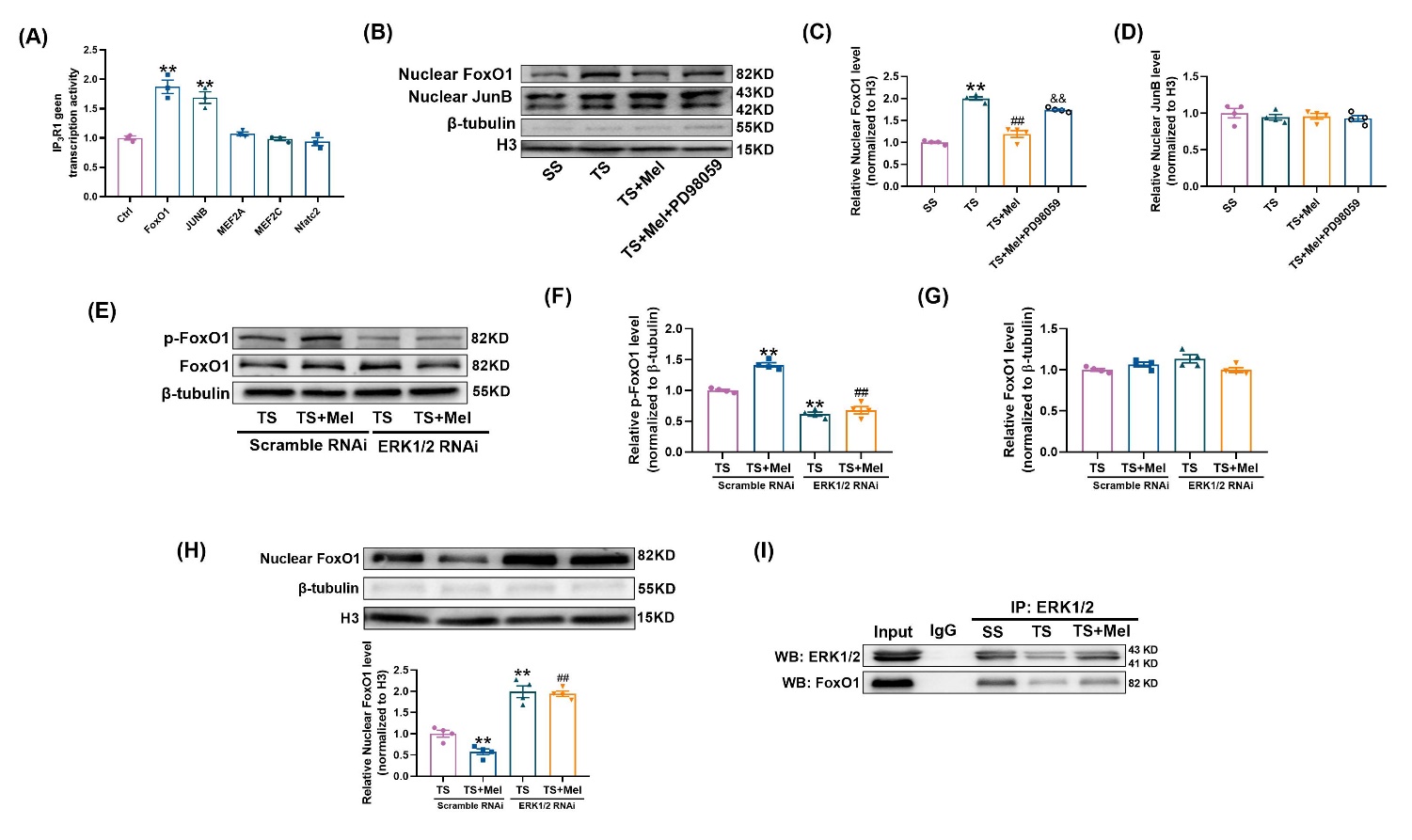
**Figure S3** Transcription factor FoxO1 rather than JUNB suppressed IP_3_R1-mediated MAMs. **(A)** FoxO1 and JUNB inhibited the luciferase activity of the IP_3_R1 promoter (n=3). **(B-D)** Representative blots and quantitative analysis of nuclear FoxO1 and JUNB (PD98059, a MEK/ERK inhibitor). **(E-H)** ERK1/2 was knocked down by siRNA, after which the cells were subjected to TS with or without melatonin. Representative blots and quantitative analysis of phosphorylated FoxO1 (p- FoxO1), total ERK1/2 and nuclear FoxO1. **(I)** Interaction between ERK1/2 and FoxO1 determined by co-immunoprecipitation (n=3). All of the values are shown as the means ± SEM. n=4 in each group. ^**^*P*< 0.01 *vs* Ctrl or SS or TS + Scramble RNAi; ^##^*P* < 0.01 *vs* TS or TS + Scramble RNAi; ^&&^*P* < 0.01 *vs* TS + Mel.


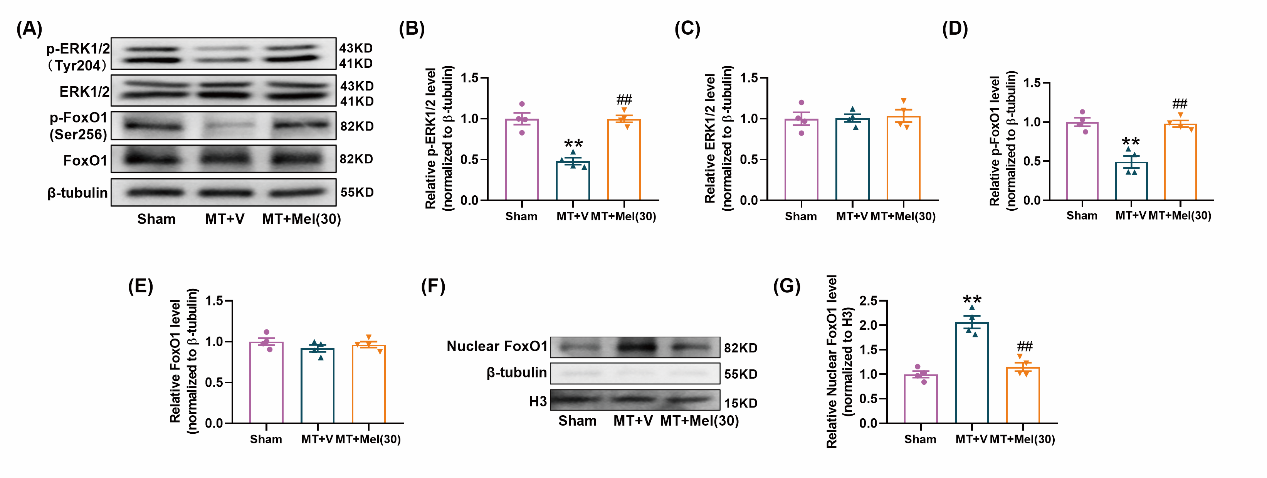
**Figure S4** Mel increased ERK1/2 and FoxO1 phosphorylation and expression of nuclear FoxO1 in MT-treated livers in vivo. **(A-G)** Representative blots and quantitative analysis of phosphorylated ERK1/2 (p-ERK1/2), ERK1/2, phosphorylated FoxO1 (p-FoxO1), FoxO1, and nuclear FoxO1; Mel, melatonin at a dosage of 30 mg/kg. n = 4 in each group. All data are shown as means ± SEM. ^**^*P*< 0.01 *vs* Sham; ^##^*P*< 0.01 *vs* MT + V.
