# Supplementary material for: IP3R1-mediated MAMs formation contributes to mechanical trauma-induced hepatic injury and the protective effect of melatonin
Source: Cell Mol Biol Lett. 2024 Feb 2;29:22. doi: 10.1186/s11658-023-00509-x (PMC10836028; doi:10.1186/s11658-023-00509-x)
Supplement: Supplementary file 2 — Additional file 2: Table S1. Primer sequences used in the study. Table S2. The antibodies used for western blotting (WB), Co-immunoprecipitation (Co-IP) and Chromatin immunoprecipitation (ChIP). [file 11658_2023_509_MOESM2_ESM.docx]

**Table S1. Primer sequences used in the study**

| Primer name | Sequence | |
| --- | --- | --- |
|  | Forward | Reverse |
| **Primer for quantitative real-time PCR in hepatocytes** | | |
| IP_3_R1 | CGTTTTGAGTTTGAAGGCGTTT | CATCTTGCGCCAATTCCCG |
| GAPDH | AGGTCGGTGTGAACGGATTTG | TGTAGACCATGTAGTTGAGGTCA |
| **Primers targeting IP_3_R1 promoter region in Chromatin immunoprecipitation analysis** | | |
| IP_3_R1 | TTTCACGAGAGGAGGCAAGG | GCCTCTAAGCCCTTAGGCAA |

**Table S2. The antibodies used for western blotting (WB), Co-immunoprecipitation (Co-IP) and Chromatin immunoprecipitation (ChIP)**

| **Antibody** | **Supplier** | **Catalog No.** | **Working dilutions** |
| --- | --- | --- | --- |
| Cleaved caspase-3 | Cell signaling  technology | #9664 | WB: 1/500 |
| IP_3_R1 | Proteintech | #19962-1-AP | WB: 1/500 |
| Mfn1 | GeneTex | #GTX133351 | WB: 1/1000 |
| Mfn2 | Abcam | #ab56889 | WB: 1/1000 |
| GRP75 | Abcam | #ab227215 | WB: 1/1000 |
| VDAC1 | Proteintech | #55259-1-AP | WB: 1/1000 |
| Bap31 | Proteintech | #11200-1-AP | WB: 1/1000 |
| Fis1 | Proteintech | #10956-1-AP | WB: 1/1000 |
| p-ERK1/2 | Proteintech | #28733-1-AP | WB: 1/1000 |
| ERK1/2 | Abcam | #ab184699 | WB: 1/1000  IP: 1/70 |
| FoxO1 | Cell Signaling Technology | #2880T | WB: 1/1000  ChIP: 1/50 |
| p-FoxO1 | Cell Signaling Technology | #9461T | WB: 1/1000 |
| JUNB | Proteintech | #10486-1-AP | WB: 1/1000 |
| H3 | Proteintech | #17168-1-AP | WB: 1/1000 |
| β-tubulin | Proteintech | #10094-1-AP | WB: 1/1000 |
| Goat Anti-Rabbit IgG Secondary Antibody | Proteintech | #SA00001-2 | WB: 1/5000 |
| Goat Anti-Mouse IgG Secondary Antibody | Proteintech | #SA00001-1 | WB: 1/5000 |
